# Inflammation as a Possible Trigger for Mitoxantrone-Induced Cardiotoxicity: An In Vivo Study in Adult and Infant Mice

**DOI:** 10.3390/ph14060510

**Published:** 2021-05-26

**Authors:** Ana Reis-Mendes, José Luís Dores-Sousa, Ana Isabel Padrão, Margarida Duarte-Araújo, José Alberto Duarte, Vítor Seabra, Salomé Gonçalves-Monteiro, Fernando Remião, Félix Carvalho, Emília Sousa, Maria Lourdes Bastos, Vera Marisa Costa

**Affiliations:** 1 UCIBIO, REQUIMTE, Laboratory of Toxicology, Department of Biological Sciences, Faculty of Pharmacy, University of Porto, 4050-313 Porto, Portugal; joseldsousa@gmail.com (J.L.D.-S.); remiao@ff.up.pt (F.R.); felixdc@ff.up.pt (F.C.); mlbastos@ff.up.pt (M.L.B.); 2 Research Center in Physical Activity, Health and Leisure (CIAFEL), Laboratory for Integrative and Translational Research in Population Health (ITR), Faculty of Sport, University of Porto, 4200-450 Porto, Portugal; apadrao@fade.up.pt (A.I.P.); jarduarte@fade.up.pt (J.A.D.); 3 LAQV/REQUIMTE, University of Porto, 4050-313 Porto, Portugal; mdcma@icbas.up.pt; 4 Department of Immune-Physiology and Pharmacology, Institute of Biomedical Sciences Abel Salazar, University of Porto, 4050-313 Porto, Portugal; 5 IINFACTS—Institute of Research and Advanced Training in Health Sciences and Technologies, University Institute of Health Sciences (IUCS), CESPU, 4585-116 Paredes, Portugal; vitor.seabra@iucs.cespu.pt; 6 LAQV/REQUIMTE, Laboratory of Pharmacology, Department of Drug Sciences, Faculty of Pharmacy, University of Porto, 4050-313 Porto, Portugal; salomemonteiro8180@gmail.com; 7 MOREHealth, Outcomes Research Lab, Portuguese Institute of Oncology at Porto Francisco Gentil (IPO Porto), 4200-072 Porto, Portugal; 8 Laboratory of Organic and Pharmaceutical Chemistry, Chemistry Department, Faculty of Pharmacy, University of Porto, 4050-313 Porto, Portugal; esousa@ff.up.pt; 9 CIIMAR—Interdisciplinary Centre of Marine and Environmental Research, 4450-208 Porto, Portugal

**Keywords:** mitoxantrone, cardiotoxicity, inflammation, oxidative stress, age, cumulative dose

## Abstract

Mitoxantrone (MTX) is a pharmaceutical drug used in the treatment of several cancers and refractory multiple sclerosis (MS). Despite its therapeutic value, adverse effects may be severe, namely the frequently reported cardiotoxicity, whose mechanisms need further research. This work aimed to assess if inflammation or oxidative stress-related pathways participate in the cardiotoxicity of MTX, using the mouse as an animal model, at two different age periods (infant or adult mice) using two therapeutic relevant cumulative doses. Histopathology findings showed that MTX caused higher cardiac toxicity in adults. In MTX-treated adults, at the highest dose, noradrenaline cardiac levels decreased, whereas at the lowest cumulative dose, protein carbonylation increased and the expression of nuclear factor kappa B (NF-κB) p65 subunit and of M1 macrophage marker increased. Moreover, MTX-treated adult mice had enhanced expression of NF-κB p52 and tumour necrosis factor (TNF-α), while decreasing interleukin-6 (IL-6). Moreover, while catalase expression significantly increased in both adult and infant mice treated with the lowest MTX cumulative dose, the expression of glyceraldehyde-3-phosphate dehydrogenase (GAPDH) and glutathione peroxidase only significantly increased in infant animals. Nevertheless, the ratio of GAPDH to ATP synthase subunit beta decreased in adult animals. In conclusion, clinically relevant doses of MTX caused dissimilar responses in adult and infant mice, being that inflammation may be an important trigger to MTX-induced cardiotoxicity.

## 1. Introduction

Mitoxantrone (MTX) is a well-established anticancer agent used in metastatic breast cancer, acute myeloid leukaemia, non-Hodgkin’s lymphoma, acute lymphoblastic leukaemia in children, metastatic hormone-refractory prostate cancer, as well as in aggressive or refractory multiple sclerosis (MS) [1]. MTX inhibits DNA and RNA synthesis through DNA intercalation and topoisomerase-II inhibition [2]. MTX is used in MS because of its powerful immunosuppressive and immunomodulatory properties, abrogating T helper cell activity, enhancing T suppressor cell function, inhibiting B lymphocyte function, and macrophage proliferation. MTX also inhibits the synthesis of pro-inflammatory cytokines, namely interferon γ (IFN-γ), tumour necrosis factor (TNF-α), interleukin-2 (IL-2), and overall antibody production [3,4].

Despite its clinical efficacy, MTX’s high lipophilicity facilitates its distribution to healthy tissues and organs, namely to the heart, where it accumulates [5], which may lead to deleterious effects in this organ. In fact, MTX-induced cardiotoxicity is one of its most severe side effects and this can hamper its therapeutic use. MTX-induced cardiac side effects increase with the total cumulative dose, so MTX recommended maximum lifetime cumulative dose has been established at 140 mg/m^2^ for cancer treatment [6] and 100 mg/m^2^ in MS treatment. Nevertheless, MTX cardiotoxicity has been described in lower cumulative doses [7]. In a study by Rivera and collaborators, cardiotoxicity was observed in patients who received cumulative doses of ≤75 mg/m^2^. Actually, 19.1% of the 97 patients enrolled have developed cardiotoxicity, with 6.19% of those receiving ≤35 mg/m^2^, 19.6% receiving 36–75 mg/m^2^, 34.0% receiving 76–105 mg/m^2^, and only 40.2% of the patients received >105 mg/m^2^ of MTX [8]. Clinical signs of MTX-inflicted cardiotoxicity include decreased left ventricular ejection fraction, heart failure (HF), and/or dilated cardiomyopathy [6].

Myocardial inflammation has emerged as a pathophysiologic process that contributes to cardiac hypertrophy, fibrosis, and dysfunction [9,10,11]. In that context, activation and nuclear translocation of the nuclear factor kappa B (NF-κB) may play an important role in the regulation of cardiac inflammatory signalling pathways, being a major contributor to myocardial dysfunction and remodelling as seen in HF [12,13]. On the other hand, oxidative stress can activate NF-κB pathways with consequent cytokine production stimulation [14,15]. Cytokines contribute to the initiation and modulation of critical responses within the overloaded myocardium, leading to myocyte growth, apoptotic myocyte death, and reactive fibrosis [16,17]. Interestingly, one of the cytokines involved, TNF-α, exerts antioxidant and antiapoptotic effects in the myocardium at physiological levels [18,19], but when levels increase, it stimulates myocyte apoptosis [20], cardiac hypertrophy, and fibrosis [21]. Another cytokine involved in cardiac homeostasis is interleukin-6 (IL-6), which possesses univocal antiapoptotic properties, protecting cardiac myocytes during acute damage [22]. However, if IL-6 levels remain chronically elevated, it promotes a sustained inflammatory response, contributing to cardiac hypertrophy [23]. It is also a strong marker of diastolic dysfunction, ejection fraction, risk, and severity of HF [23]. Despite the MTX’s immunomodulatory effect on MS, to the best of our knowledge, its role on inflammatory cardiac pathways has not been traced yet. 

Noteworthy, an important factor that influences the development of MTX cardiotoxicity is patients’ age. Available studies demonstrate that the very young and the elderly are more prone to develop cardiotoxicity after MTX-treatment [8,24], but the effect on adults also has an important social and economic impact, especially in a moment where life expectancy is increasing. Several studies have also shown that MTX is able to trigger cardiotoxicity in small children (<4 years) [25,26,27]. These effects can last and affect their life expectancy and quality of life [28,29,30,31]. This factor must be put into perspective nowadays, as prior to the 1970s, the 5-year survival of childhood cancer was lower than 50% [32], while now it is reaching 85% [33]. Therefore, the number of children who survive cancer is drastically increasing, being that chemotherapy side effects, such as cardiotoxicity, will have a long-term impact. Nevertheless, the reasons for age susceptibility towards MTX-inflicted cardiotoxicity have been poorly investigated [34].

This study aimed to evaluate MTX-induced cardiotoxicity in two groups of animals of different age periods (infant and adults) and two cumulative doses. The highest cumulative dose was used as a starting point to assess the mechanisms of cardiotoxicity induced by MTX. The lowest cumulative dose caused less animal suffering but allowed for the assessment of MTX interference towards inflammation or oxidative stress pathways, and to determine early markers of heart damage that allows the study of subtle and even non-clinically undisclosed cardiotoxic mechanisms. 

## 2. Results

**Figure 1:**
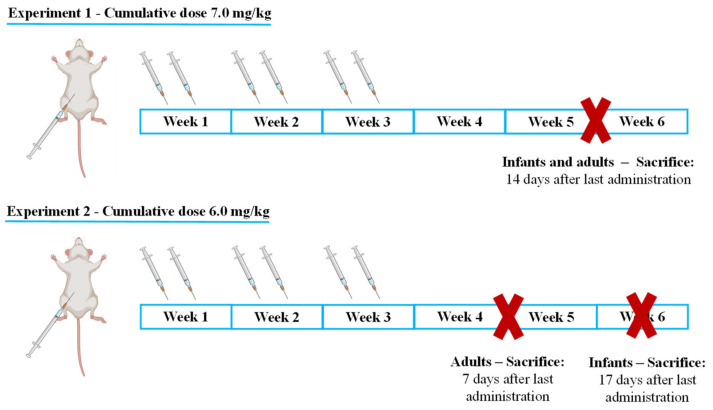
Schematic representation of the distribution of the animals by cumulative dose, as well as the timeline of administration of MTX and sacrifice.

In experiment 1, both adults and infants received a 7.0 mg/kg cumulative dose and were sacrificed 14 days after the last administration (Figure 1). The results obtained in experiment 1 together with previously published data [34] indicate that infants are more resilient to MTX side effects, as will be shown in this section. As suffering was apparent in most of the animals that received the highest dose, in experiment 2, the dose was decreased (6.0 mg/kg) (Figure 1), and two different approaches were taken:

- Infant mice were sacrificed 17 days after the last administration to determine if, with increased lag time, infants would present signs of cardiac damage or still maintained higher cardiac resilience;
- Adults were sacrificed earlier, namely after 7 days to determine early markers of heart damage that may reveal subtle cardiotoxic mechanisms before unacceptable damage occurred.

### 2.1. MTX Caused Significant Effects on Body Weight and Food/Water Consumption

In the infant population, 7.0 mg/kg MTX-treated animals had significantly less body weight gain when compared to controls after the 17th day (Figure S1A). The 7.0 mg/kg MTX-treated adult mice had a lower average body weight than the control group after reaching the maximum cumulative dose. In detail, statistically significant differences in body weight occurred after the 21st day, and loss of weight occurred on the 24th day (Figure S1B).

The amount of food and water intake was based on each animal’s body weight, since the animals were maintained in a social environment and therefore in a group. Regarding water consumption, overall, the 7.0 mg/kg MTX-treated infants (Figure S1C) and adults (Figure S1D) had lower consumption than controls during all the experimental periods. Food consumption was significantly decreased in the case of the 7.0 mg/kg MTX-treated infant group (Figure S1E), after the 14th day. Adults that received the cumulative dose of 7.0 mg/kg had statistically significant higher food consumption in the week following the last MTX administration, compared with the control group (Figure S1F).

In the infant population, animals had initially significant differences in the body weight gain between the 6.0 mg/kg MTX-treated and the control groups (Figure S2A). These initial differences were due to the random distribution, for each cage, of the mice belonging to different litters (Figure S2A). On the other hand, in the 6.0 mg/kg MTX-treated adult population, a statistically significant body weight decrease was observed in the last days of the experiment (Figure S2B).

Concerning water intake, a decrease in MTX-treated infant population intake was observed throughout the experiment, reaching statistical significance by the end of the protocol, when compared with the control group (Figure S2C). Regarding the adults treated with a MTX cumulative dose of 6.0 mg/kg, a statistically significant decrease in water intake was observed in the last days of the experiment (Figure S2D). Similar to what happened in water intake, food consumption in the 6.0 mg/kg MTX-treated infant mice decreased, being statistically significant in the last days of the protocol, when compared with the control group (Figure S2E). We found that a significant decrease between the food consumption of the MTX 6.0 mg/kg-treated adults and the control group was observed towards the end of the experiment (Figure S2F). 

### 2.2. Plasma Levels of Total-CK and AST/ALT Ratio Were Increased in the 7.0 mg/kg MTX-Treated Adult Mice

In the 7.0 mg/kg MTX-treated infant group, no statistically significant differences were observed in the aminotransferase’s levels, when compared to control mice (Table S2). Concerning the adult mice, a tendency for increased aspartate aminotransferase (AST)/alanine aminotransferase (ALT) ratio of the 7.0 mg/kg MTX-treated group, compared with the control, was observed (*p* = 0.06). In the 6.0 mg/kg MTX-treated infants, ALT levels were elevated while the AST/ALT ratio was found to be decreased when compared with the control group. Concerning the 6.0 mg/kg-treated adult mice, there were no significant changes in plasma aminotransferases (Table S3).

In the infant mice, plasma creatine kinase–MB (CK–MB) levels were significantly higher in the 7.0 mg/kg MTX-treated mice when compared with the respective controls (Table S2), while no statistically significant changes were observed in the plasma total creatine kinase (total-CK) levels. In adult mice, plasma total-CK levels were significantly higher in the 7.0 mg/kg MTX-treated mice, compared to respective controls (Table S2), but no meaningful changes were found in their plasma CK–MB values compared to controls. No significant differences in CK–MB and total-CK levels were found between control and the 6.0 mg/kg MTX-treated adult animals (Table S3). Unfortunately, in the 6.0 mg/kg-treated infant mice, total-CK and CK–MB levels were not determined due to an insufficient amount of plasma. 

### 2.3. The Ratio of Heart/Body Weight Decreased in Adults after the Highest Dose of MTX

Heart to body weight ratio was significantly decreased in the 7.0 mg/kg MTX group (Table S2). In the 6.0 mg/kg MTX-treated adult population, no significant differences were observed in heart weight/body weight ratio (Table S3). In addition, no statistically significant differences were observed in heart/body weight ratio between MTX-treated infant mice and the control group, in both doses (Tables S2 and S3).

### 2.4. Histological Damage Occurred in Cardiac Tissue after MTX-Treatment, Adult Population Being More Susceptible to Cardiac Damage

**Figure 2:**
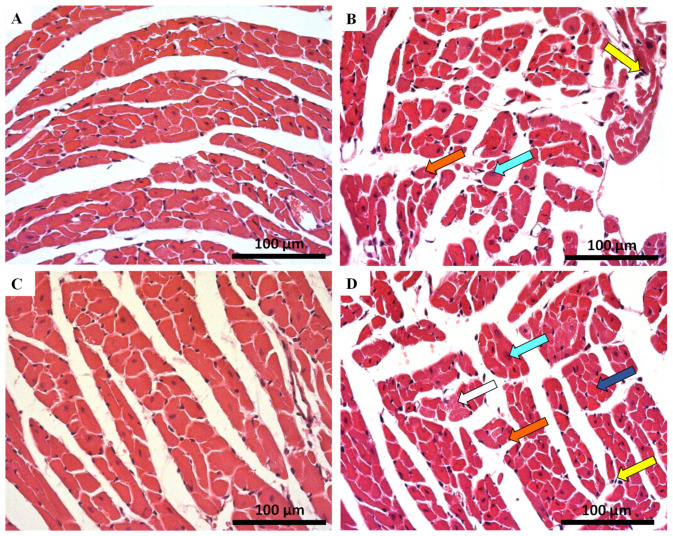
Cardiac histopathology evaluation done by light microscopy from MTX-treated adult and infant animals and respective controls, as assessed by haematoxylin and eosin staining (experiment 1, mice were euthanized 14 days after the last administration) (**A**–**D**). Light micrograph from: (**A**) infant mice controls in experiment 1, showing normal morphology and structure; (**B**) infant mice given a cumulative dose of 7.0 mg/kg MTX; (**C**) adult mice in experiment 1 showed normal morphology and structure; (**D**) adult mice given a cumulative dose of 7.0 mg/kg MTX. Presence of vacuolization (orange arrow), inflammatory infiltration (yellow arrow), as well as large and uncondensed nucleus (cyan arrow). Cellular oedema (white arrow) and necrotic zones (blue arrow) are evident only in adult mice. Scale bar = 100 µm, *n* = 3. Images taken at 40× amplification.

**Figure 3:**
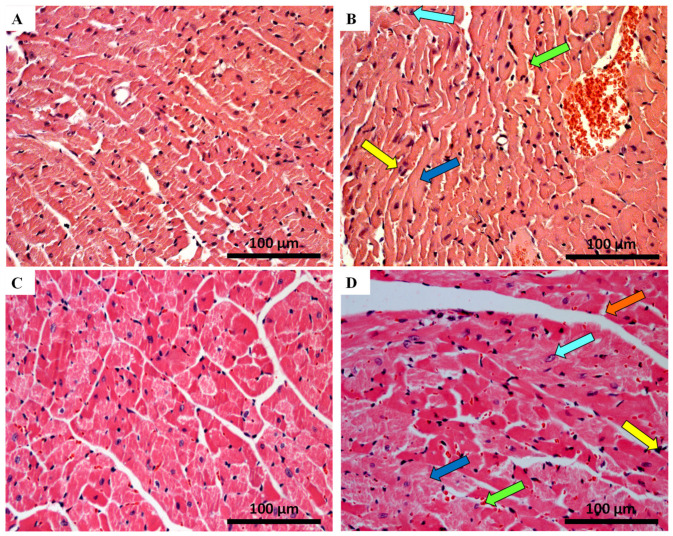
Cardiac histopathology evaluation done by light microscopy from MTX-treated adult and infant animals and respective, as assessed by haematoxylin and eosin staining controls (experiment 2, adult and infant mice were euthanized 7 or 17 days after the last administration, respectively) (**A**–**D**). Light micrograph from: (**A**) infant mice controls in experiment 2, showing normal morphology and structure; (**B**) infant mice given a cumulative dose of 6.0 mg/kg MTX; (**C**) adult mice in experiment 2 showed normal morphology and structure; (**D**) adult mice given a cumulative dose of 6.0 mg/kg MTX. Presence of vacuolization (orange arrow), inflammatory infiltration (yellow arrow), vascular congestion (green arrow), necrotic zones (blue arrow), as well as large and uncondensed nucleus (cyan arrow) is evident. Scale bar = 100 µm, *n* = 3. Images taken at 40× amplification.

The qualitative histologic examination of the heart of MTX-treated mice and controls was done using the haematoxylin and eosin staining. Major qualitative and representative structural alterations are depicted in Figure 2 and Figure 3. Meanwhile, the results of the semi-quantitative analysis are presented in Table 1. Lesions in the cardiac tissue caused by MTX were characterized by cellular degeneration, interstitial inflammatory cell infiltration, and necrotic zones. In experiment 1, the heart of both infant and adult control mice showed a preserved heart tissue structure (Figure 2A,C). In the 7.0 mg/kg MTX-treated infant group, cardiac tissue showed interstitial inflammatory cell infiltration, signs of vacuolization, as well as necrotic zones but to a lower extent when compared with adults. In the 7.0 mg/kg MTX-treated adult mice, the presence of cellular oedema, cytoplasmic vacuolization of cardiomyocytes, interstitial inflammatory cell infiltration, as well as some necrotic zones were evident (Figure 2D).

In experiment 2, control groups did not present any changes in the tissue structure, as expected (Figure 3A,C). The 6.0 mg/kg MTX-treated infant group showed signs of vascular congestion, with enlarged blood vessels, necrotic zones, inflammatory infiltrations, vacuolization, with a large and uncondensed nucleus (Figure 3B). In MTX-treated adults, the presence of cytoplasmic vacuolization of cardiomyocytes, vascular congestion, interstitial inflammatory cell infiltration, as well as some necrotic zones was evident (Figure 3D, Table 1). The lesions were most notorious in the endocardium. Signs of obstruction in some small calibre arteries were observed in the 6.0 mg/kg MTX-treated adult mice (data not shown). All groups showed myocardium with preserved structure (score = 0), therefore data regarding tissue disorganization were omitted from Table 1 (data not shown).

### 2.5. Myocardial Fibrosis Was Significantly Increased in MTX-Treated Adult Mice at Both Doses

**Figure 4:**
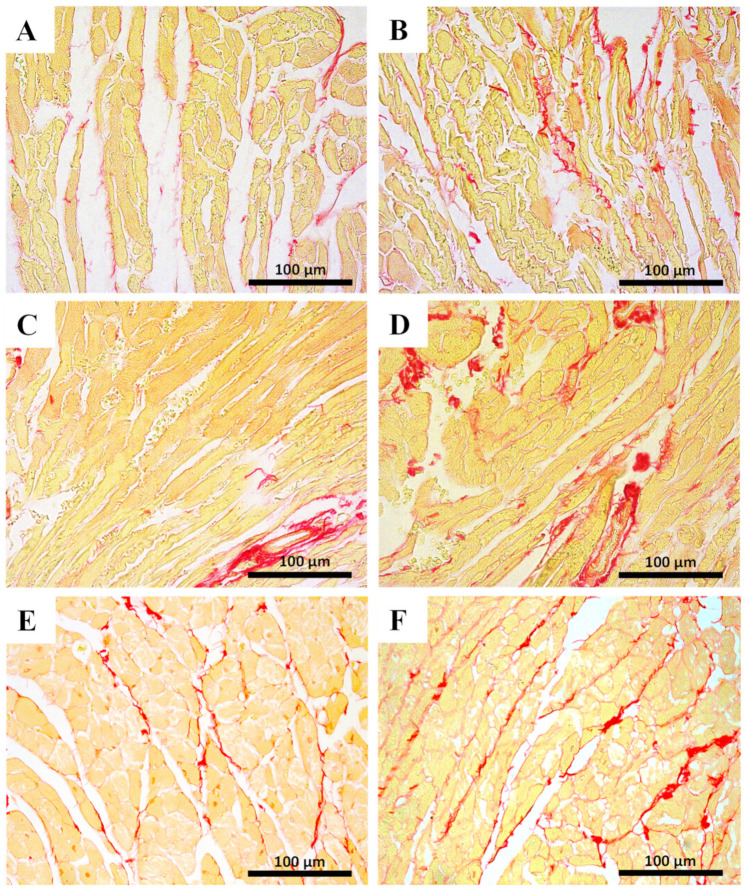
Sirius red cardiac staining assessed by light microscopy from MTX-treated adult and infant animals and respective controls (**A**–**F**). Light micrograph from (**A**) the control infant mice (experiment 1, mice were euthanized 14 days after the last administration); (**B**) infant mice injected with a cumulative dose of 7.0 mg/kg MTX; (**C**) control adult mice; (**D**) adult mice injected with a cumulative dose of 7.0 mg/kg MTX showing higher fibrosis. (**E**) Control adults (experiment 2, mice were euthanized 7 days after the last administration); (**F**) adult mice after receiving a cumulative dose of 6.0 mg/kg MTX. Scale bar = 100 µm, *n* = 3. Images taken at 40× amplification.

Myocardial fibrosis, which is a hallmark of cardiac hypertrophy, was assessed by the Sirius red technique. Results are presented in Figure 4 and Table 2. No significant differences were observed among the infant groups regarding the percentage of collagen/muscle area of the heart. In contrast, the MTX-treated adults had significant increases in the percentage of collagen/muscle area, at both doses.

### 2.6. The Highest Cumulative Dose of MTX Increased Cardiac Total Glutathione in Infants, While Decreasing Noradrenaline Cardiac Levels in Adults

The 7.0 mg/kg MTX-treated infant group presented significantly higher cardiac values of total glutathione (tGSH) than the control group (Table 3). No differences were found in oxidized glutathione (GSSG) and reduced glutathione (GSH)/GSSG ratio of the infant population treated with MTX when compared to controls. No statistically significant changes in the cardiac levels of tGSH, GSSG, and GSH/GSSG ratio were found in 7.0 mg/kg MTX-treated adult mice. In the 6.0 mg/kg MTX-treated infant and adult mice, no differences were found between groups (data not shown). Moreover, no statistically significant changes were found in the marker of lipid peroxidation, malondialdehyde (MDA), between groups in experiment 1.

In experiment 1, to understand if MTX had any impact on the cellular energetics on the two populations (infant and adult), intracellular adenosine 5′-triphosphate (ATP) and phosphocreatine levels were measured (Table 3). In the 7.0 mg/kg MTX condition in both populations, no significant differences in ATP levels were observed. Furthermore, no significant differences were seen in phosphocreatine and creatine levels in the MTX-treated infant group, when compared to control mice. However, in the adult population, there is a tendency for increased phosphocreatine levels in the MTX-treated group, compared with the control group (*p* = 0.06).

In experiment 2, no significant changes in ATP levels were observed in 6.0 mg/kg-treated mice of both ages (data not shown).

For experiment 1, levels of noradrenaline are depicted in Table 3. Noradrenaline cardiac levels were significantly lower in MTX-treated adults when compared to controls. No meaningful differences were seen between control and infant MTX-treated mice at 7.0 mg/kg.

### 2.7. The Expression of GAPDH Increased in the 6.0 mg/kg MTX-Treated Infant Mice

After major and general data were gathered, the animals treated with a lower cumulative dose (6.0 mg/kg) were subjected to other determinations as to assess early markers of damage in adults, or to determine the underlying reasons that make infants more resilient to MTX-cardiac effects after a longer washout time.

**Figure 5:**
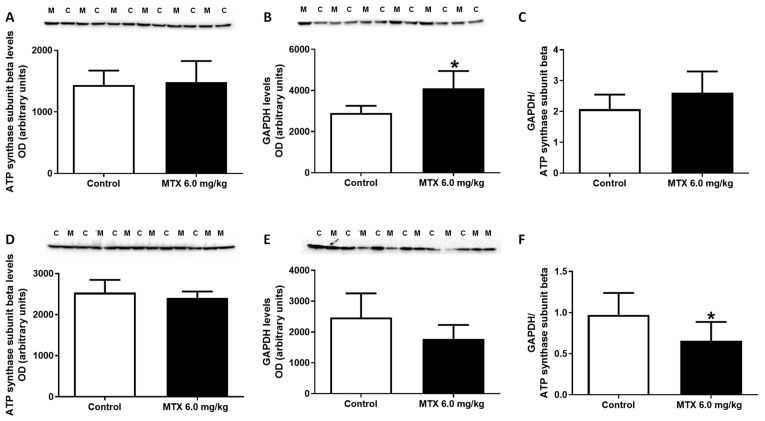
ATP synthase subunit beta (52 kDa), glyceraldehyde-3-phosphate dehydrogenase (GAPDH) (37 kDa) expression, and ratio GAPDH to ATP synthase subunit beta evaluated by Western blotting, from (**A**–**C**) infant MTX-treated (M), and control mice (C), and (**D**–**F**) adult mice exposed to a cumulative dose of 6.0 mg/kg MTX (M), and control (C) adult mice (experiment 2, infant and adult mice were euthanized 17 or 7 days after the last administration, respectively). Values are expressed as mean ± SD and were obtained from 6 (infant) and 6–7 (adult) animals from each treatment group. Statistical comparisons were made using the t-test: * *p* < 0.05, MTX 6.0 mg/kg vs. control. OD: optic density. Protein loading was confirmed by the Ponceau S staining (Figures S3 and S4).

In order to assess the impact of MTX treatment on cardiac metabolism, the expression of metabolic enzymes was evaluated by Western blotting (Figure 5). No changes were observed on the levels of ATP synthase subunit beta while the expression of the glycolytic enzyme, glyceraldehyde-3-phosphate dehydrogenase (GAPDH), was significantly higher in MTX-treated infant mice, compared with control mice (Figure 5A,B). No changes were observed in the ratio of GAPDH to ATP synthase subunit beta in MTX-treated infant mice, when compared with control mice (Figure 5C). No changes were seen in the levels of ATP synthase subunit beta nor in the levels of GAPDH in MTX-treated adult mice, when compared with control mice (Figure 5D,E). Nevertheless, significant differences were observed in the ratio of GAPDH to ATP synthase subunit beta between MTX-treated adult mice and the control group (Figure 5F).

### 2.8. At the Lower Cumulative Dose, Protein Carbonylation Increased in Adult Mice

**Figure 6:**
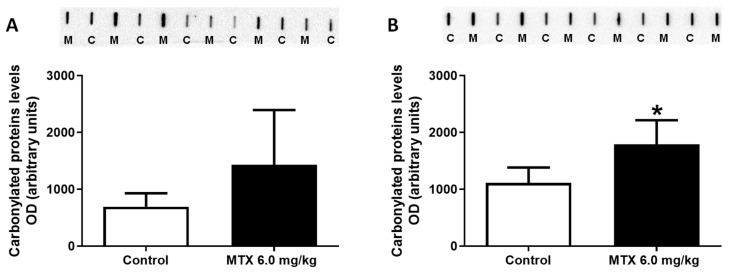
Protein carbonylation cardiac content evaluated by slot blot in (**A**) infant mice and (**B**) adult mice exposed to a cumulative dose of 6.0 mg/kg MTX (experiment 2, infant and adult mice were euthanized 17 or 7 days after the last administration, respectively). Values are expressed as mean ± SD and were obtained from six animals from each treatment group. Statistical comparisons were made using the t-test: * *p* < 0.05, MTX 6.0 mg/kg vs. control. OD: optic density, M: mitoxantrone, C: control.

The protein carbonylation in the cardiac lysates increased in the 6.0 mg/kg MTX-treated adult mice, compared to control mice (Figure 6B). No differences were found in the MTX-treated infant mice (Figure 6A).

### 2.9. MTX Increased Catalase Expression Both in Adults and Infants, While in Infants’ Glutathione Peroxidase Expression Increased

**Figure 7:**
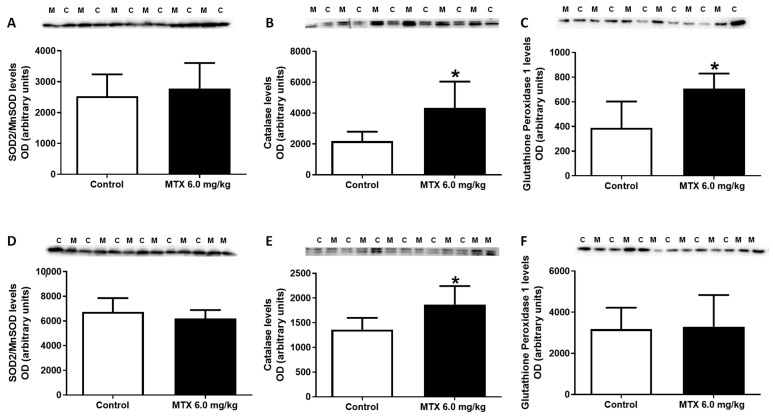
Superoxide dismutase 2/ manganese-dependent superoxide dismutase (SOD2/MnSOD) (26.6 kDa), catalase (60 kDa) and glutathione peroxidase (22 kDa) expression evaluated by Western blotting, from (**A**–**C**) infant MTX-treated (M) and control (C) mice, and (**D**–**F**) adult mice exposed to a cumulative dose of 6.0 mg/kg MTX (M) and their respective controls (C) (experiment 2, infant and adult mice were euthanized 17 or 7 days after the last administration, respectively). Values are expressed as mean ± SD and were obtained from 6 (infant) and 6–7 (adult) animals from each treatment group. Statistical comparisons were made using the t-test: * *p* < 0.05, MTX 6.0 mg/kg vs. control. OD: optic density. Protein loading was confirmed by the Ponceau S staining (Figures S3 and S4).

As shown in Figure 7C, levels of glutathione peroxidase were increased in MTX-treated infant mice, when compared with control mice, while in adults no statistically significant differences were observed (Figure 7F). Neither in adults nor infants, superoxide dismutase/ manganese-dependent superoxide dismutase (SOD2/MnSOD) had any significant changes after MTX administration (Figure 7A,D). On the contrary, the levels of catalase were increased in both MTX-treated adult and infant mice, when compared with control mice (Figure 7B,E).

### 2.10. The Heart of MTX-Treated Adult Mice Showed a Higher Density of Infiltrating M1 Macrophages

**Figure 8:**
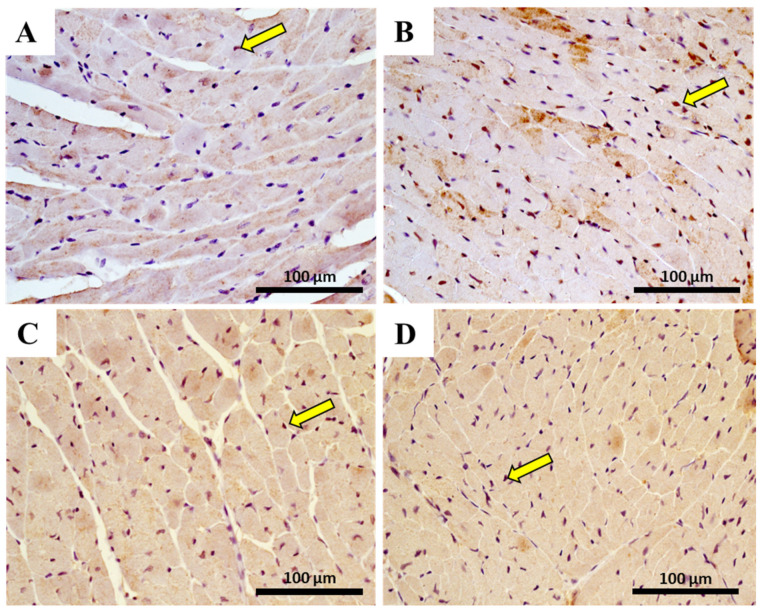
Representative photomicrographs of heart immunohistochemistry from 6.0 mg/kg MTX-treated adult and respective controls (experiment 2, mice euthanized 7 days after the last administration), by a marker (CD68+) for macrophages M1 (**A**,**B**) and by a marker for (CD206) macrophage M2 (**C**,**D**) detection, and indicated by yellow arrows. Light micrograph (**A**,**C**) from the control of M1 (**A**) and M2 staining (**C)**; light micrograph (**B**,**D**) from adult mice given a cumulative dose of 6.0 mg/kg MTX and performed staining for M1 (**B**) or M2 (**D**). Scale bar = 100 µm, *n* = 3. Images taken at 40× amplification.

The immunohistochemistry evaluation in the heart of pro-inflammatory M1 macrophages, using CD68+ immunostaining, and anti-inflammatory M2 macrophage, using CD206 immunostaining, is shown in Figure 8 and Table 4. Data analysis indicated that the number of CD68 positive cells had a significant increase (*p* < 0.001) after MTX exposure in adult mice (Table 4). Conversely, no meaningful differences were observed in the M2 subset of the adult population treated with MTX when compared with controls. In the 6.0 mg/kg MTX-treated infant group, no differences were observed in the M1 and M2 macrophages when compared with controls (photomicrographs not shown).

### 2.11. In Adults, the Lower Cumulative Dose of MTX Significantly Increased the Expression of NF-ĸB p65

**Figure 9:**
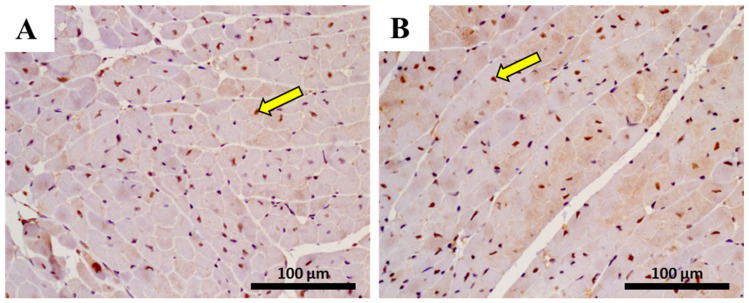
Representative photomicrographs of immunohistochemistry determination of NF-κB p65 in the cardiomyocytes-like cells from 6.0 mg/kg MTX-treated adult and respective controls (experiment 2, mice euthanized 7 days after the last administration) (**A**,**B**). Light micrograph (**A**) from the controls; and (**B**) from adult mice given a cumulative dose of 6.0 mg/kg MTX. Scale bar = 100 µm, *n* = 3. Images taken at 40× amplification.

Cardiac immunohistochemistry analysis was performed in order to assess the activation of the inflammatory transcriptional factor, NF-κB, namely the fraction p65. The immunohistochemistry data of NF-κB is shown in Figure 9 and Table 5. Immunohistochemical staining of NF-κB p65 in the control group showed low cytoplasmic expression (Figure 9A). However, the 6.0 mg/kg MTX-treated adult group showed positive cytoplasmic and nuclear expression. In addition, in MTX-treated animals, the endothelium of the blood vessels showed a high expression of p65, which was evident from the intense brown staining (Figure 9B). When performing a semiquantitative analysis, a statistically significant increase (*p* < 0.01) in the mean number of the NF-κB immunopositive cells in the MTX adult group was observed when compared with the control group (Table 5). 

Conversely, no meaningful differences were observed in the infant population treated with MTX when compared with controls (photomicrographs not shown). 

**Figure 10:**
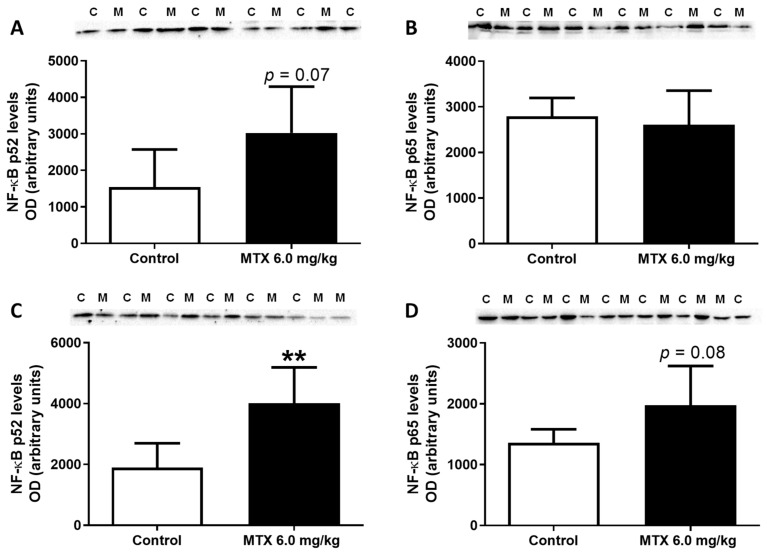
Nuclear kappa B nuclear transcription factor (NF-κB) p52 (50 kDa) and NF-κB p65 (60 kDa) expression evaluated by Western blotting, from (**A**,**B**) infant MTX-treated (M) and control (C) mice, and (**C**,**D**) adult mice exposed to a cumulative dose of 6.0 mg/kg MTX (M) and respective controls (C) (experiment 2, infant and adult mice were euthanized 17 or 7 days after the last administration, respectively). Values are expressed as mean ± SD and were obtained from 5–6 (infant) and 6–7 (adult) animals from each treatment group. Statistical comparisons were made using the t-test: ** *p* < 0.01, MTX 6.0 mg/kg vs. control. OD: optic density. Protein loading was confirmed by the Ponceau S staining (Figures S3 and S4).

Subsequently, Western blot analysis showed that, in the cardiac total homogenates, MTX led to a tendency for increased expression of NF-κB p65 levels (*p* = 0.08) (Figure 10D), while no differences were observed in the infant mice (Figure 10B). Furthermore, MTX-treated adult mice had enhanced expression of NF-κB p52 (Figure 10C), while MTX-treated infant mice showed a tendency for increased NF-κB p52 levels (*p* = 0.07) in comparison with the control group (Figure 10A).

### 2.12. MTX Increased TNF-α Cardiac Expression while it Decreased IL-6

**Figure 11:**
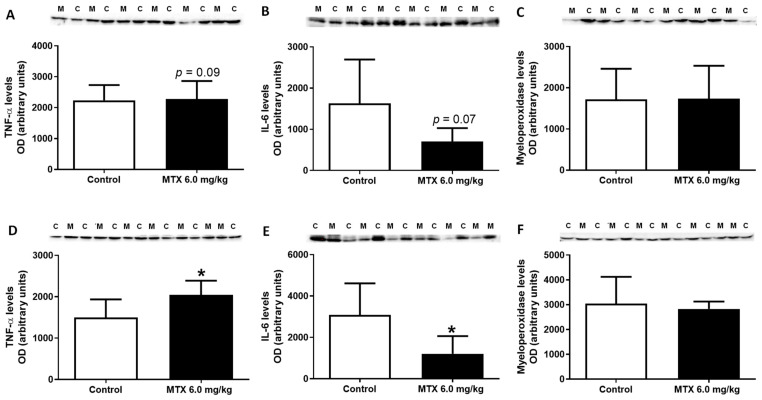
Tumour necrosis factor-α (TNF- α) (25 kDa), interleukin-6 (IL-6) (24 kDa), myeloperoxidase (48 kDa) expression evaluated by western blotting, from (**A**–**C**) infant MTX-treated (M) and control (C) mice, and (**D**–**F**) adult mice exposed to a cumulative dose of 6.0 mg/kg MTX (M) and respective controls (C) (experiment 2, infant and adult mice euthanized 17 or 7 days after the last administration, respectively). Values are expressed as mean ± SD and were obtained from 6 (infant) and 6–7 (adult) animals from each treatment group. Statistical comparisons were made using the t-test: * *p* < 0.05, MTX 6.0 mg/kg vs. control. OD: optic density. Protein loading was confirmed by the Ponceau S staining (Figures S3 and S4).

TNF-α was evaluated and MTX-treated adult mice had a significant increase in its expression in comparison with the control group (Figure 11D), while MTX-treated infant mice showed a tendency for increased TNF-α levels (*p* = 0.09) in comparison with the control group (Figure 11A).

Conversely, MTX significantly decreased IL-6 levels in the heart of MTX-treated adult mice (Figure 11E), while the MTX-treated infant mice only showed a tendency for decreased IL-6 levels (*p* = 0.07) in comparison with the respective control group (Figure 11B). 

Finally, data showed that MTX did not alter the expression of myeloperoxidase in both populations (Figure 11C,F).

## 3. Discussion

The major findings of this work were: (1) MTX treatment affected animals’ body weight and food/water consumption; (2) histopathological examination showed that all MTX-treated groups had cardiac injury, being more severe in adults, and only adults had a significant increase in fibrotic tissue, in both MTX doses; (3) infants showed some adaptation features after MTX, namely the highest cumulative dose of MTX led to increased tGSH levels, while the expression of GAPDH and glutathione peroxidase only increased in 6.0 mg/kg MTX-treated infant animals; (4) the lower cumulative dose (6.0 mg/ kg) elicited an increase in catalase expression, in both adult and infant mice; (5) in adults, noradrenaline cardiac levels decreased after 7.0 mg/kg MTX, whilst at the lower cumulative dose, protein carbonylation and the expression of NF-κB p65 and p52 subunits, and M1 macrophage marker increased; (6) TNF-α and IL-6 were differentially expressed within the myocardium in response to administration of MTX 6.0 mg/kg. 

In experiment 1, the 7.0 mg/kg MTX-treated adult group had significantly less body weight compared to controls, whereas changes in body weight in the infant population were only seen after the 17th day. In fact, in experiment 2, the lowest dose of 6.0 mg/kg of MTX also caused a statistically significant body weight decrease in the adult population after the 21st day. We observed that, in general, food and water intake decreased in the following days after MTX-administration. Several studies reported a loss of body weight in mice after MTX administration [34,35,36,37]. Signs of diarrhoea after the last administration of MTX in adults were seen, but not in infants, and therefore this fact can contribute to the weight decrease. In humans, MTX induces gastrointestinal alterations, such as nausea, vomiting, diarrhoea, and mucositis [6]. 

Plasma AST, ALT, total-CK, and CK–MB levels were measured in animals of both experiments. In experiment 1, no significant differences in the aminotransferase levels (AST and ALT) were found. However, a tendency for an increase was observed in the AST/ALT ratio of the 7.0 mg/kg MTX-treated adult population. An increased AST/ALT ratio has been proposed as a marker for the assessment of heart damage [38]. Dores-Sousa et al. also showed that animals sacrificed 24 h after the last MTX administration (9.0 mg/kg cumulative dose given in six administrations) had no significant differences in the aminotransferase levels in any of the MTX-treated groups [34]; nevertheless, three weeks after the last administration, AST/ALT ratio increased in the remaining infant mice, suggesting heart damage [34]. In experiment 2, only the 6.0 mg/kg-treated infant population had elevated ALT levels, while the AST/ALT ratio was found to be significantly decreased. Other authors reported increased ALT levels in rats treated with a cumulative dose of 7.5 mg/kg of MTX [39]. Therefore, the classic markers of heart damage were not a good correlation to the damage seen in this lower dose, either in earlier time point in adults or later time point in infants.

Regarding total-CK and CK–MB, in experiment 1, the 7.0 mg/kg cumulative MTX dose induced a significant elevation in plasma CK–MB in infant mice, which may be indicative of recent cardiac damage. In another study, mice treated with MTX [three doses of 2.5 mg/kg MTX (intraperitoneal (IP))], had increased serum levels of CK–MB [40]. In addition, the total-CK values of the 7.0 mg/kg MTX-treated adults were significantly higher than controls, which suggests that the overall muscle activity apparently is changed by MTX. No significant differences were seen in the 6.0 mg/kg MTX mice. Conversely, the heart weight/ body weight ratio decreased in MTX-treated adult mice at a cumulative dose of 6.0 mg/kg. On the contrary, an increase in cardiac weight in rats 28 days after administering three doses of 2.5 mg/kg MTX was reported [37], suggesting different adaptation stages.

MTX treatment resulted in cardiac tissue damage manifested by histopathological changes. The histological examination of both right and left ventricles of the 7.0 mg/kg MTX-treated heart revealed cellular oedema, necrotic zones, signs of vacuolization, interstitial inflammatory cell infiltration, all signs of cellular degeneration. In the 6.0 mg/kg MTX-treated mice, signs of vascular congestion were observed, with enlarged blood vessels, inflammatory infiltrations in the interstitial spaces of the cardiomyocytes, and proliferation of connective tissue, large and uncondensed nucleus, the presence of cytoplasmic vacuolization, as well as some necrosis. Signs of obstruction in some small calibre arteries were observed in the 6.0 mg/kg MTX-treated adult mice. These changes were more evident in the sub-endocardial region when compared with the sub-pericardial one. According to the published literature, MTX produces degenerative cardiomyopathy evidenced by micro- and macro-vacuolization, intercellular oedema, atrophy, inflammatory infiltrates, aberrant mitochondria, myolysis of cardiomyocytes, and interstitial fibrosis in rodents [34,37,41,42], and humans [43]. These features were also observed herein, thus highlighting the relevance of our model. In the semi-quantitative histological analysis, the changes in heart tissue in infant mice occurred at a lower extent than in adults, in both cumulative doses. While one would expect that cardiac plasma toxicity markers would be increased in both studied populations, since histological damage was observed, that did not occur in the majority of the determinations. Whether the clearance of those markers from plasma, because of earlier damage or if the biomarkers are not sensitive enough for the damage endured, is not clear at this point. 

The sympathetic and adrenal systems are key for cardiac function, and noradrenaline is the main neurotransmitter/hormone responsible for the rapid adjustment of the heart to the body’s needs. In infant mice, the cardiac levels of noradrenaline did not suffer any significant changes, while adults treated with 7.0 mg/kg MTX had lower levels of noradrenaline than controls. In a previous work by the group, a cumulative dose of 9.0 mg/kg of MTX also caused a statistically significant decrease in cardiac noradrenaline levels in adults, suggesting that MTX causes noradrenergic dysfunction in adult animals [34], while not affecting infants. Age-associated changes in the autonomic nervous system are linked to increased cardiac sympathetic tone with decreased parasympathetic input, dampened cardiovagal baroreceptor sensitivity, and reduced clearance of plasma noradrenaline [44], which seems to be exacerbated by MTX. 

Another crucial aspect of cardiac homeostasis relates to the hearts’ energetic balance. The primary source of energy in the heart is phosphocreatine, which maintains local ATP pools and stabilizes cellular membranes due to electrostatic interactions with phospholipids [45]. Phosphocreatine has the ability to stabilize membranes and protect cells from damage [46]. In experiment 1, in the adult population treated with 7.0 mg/kg of MTX, a tendency existed for increased phosphocreatine levels, while no significant changes of creatine and phosphocreatine levels in infant mice were seen. The increased phosphocreatine seen in the MTX-treated adults may be an attempt to protect the cells or to stimulate protein synthesis [46]. It is clear that the phosphocreatine system is important to promote de novo synthesis of ATP, by means of CK action [47]. However, no changes in cardiac ATP were verified in the heart of both MTX-treated mice compared to controls. Similar results were reported by others, namely with a cumulative dose of 9.0 mg/kg, when animals were sacrificed 24 h after the last administration [34] or at a 7.5 mg/kg cumulative dose and sacrificed 2 days after the last administration [37]. Nevertheless, in the work of Rossato et al., when rats were administered with the cumulative dose of 7.5 mg/kg MTX but sacrificed 28 days after the last administration, a significant depletion of cardiac ATP levels took place, suggesting that ATP depletion is a late event [37], possibly because response/adaptation towards insult is probably overcome at that stage. The heart consumes large amounts of energy in the form of ATP that is continuously replenished by oxidative phosphorylation in mitochondria and, to a lesser extent, by glycolysis. Despite unchanged ATP synthase subunit beta levels, the expression of the glycolytic enzyme GAPDH was significantly higher in the MTX-treated infant mice (6.0 mg/kg), while glycolytic ratio (GAPDH/ATP synthase subunit beta) changes were seen in adults sacrificed earlier, suggesting that although the infant mice are more resilient to the highest dose, later on (17 days) a metabolic shift to glucose oxidation occurs, which may be a determinant sign of damage. A shift from the oxidation of fatty acids to increased oxidation of glucose, along with the inhibition of OXPHOS activity, has been described for another topoisomerase IIb inhibitor, doxorubicin, being these key features of heart damage [48]. Recently, our group has shown through a proteomic study in mitochondrial enriched fractions that MTX also causes downregulation of the fatty oxidation metabolic process [49]. 

Although MTX has been considered with a low ability to induce oxidative stress, several studies have been focused on those markers when MTX heart damage is being researched [34,37,50]. While in adults given a 7.0 mg/kg cumulative dose of MTX, no significant changes in the cardiac levels of the tGSH, GSSG, and GSH/GSSG ratio were found, in the infants that took the same cumulative dose, cardiac tGSH levels increased significantly. Dores-Sousa et al. had similar results when 9.0 mg/kg of MTX was administered to infant mice that presented the highest values of tGSH three weeks after the last administration. The authors suggested that there was an adaptation response of that population related to an up-regulation of γ-glutamylcysteine synthetase (γ-GCS) activity in the younger population [34]. In that sense, a recent work of the group showed that cardiac HL-1 cells exposed to MTX also had higher levels of tGSH, and that this increase was abrogated by the γ-GCS inhibitor, buthionine sulfoximine. On the other hand, in the 6.0 mg/kg MTX-treated infant and adult animals, in experiment 2, no differences in tGSH and GSSG values or ratios were observed; similarly, other authors also reported no statistically significant changes in the cardiac GSH levels in animals treated with an acute dose of 15.0 mg/kg of MTX, 4 days after administration [50], or with a cumulative dose of 7.5 mg/kg of MTX, 28 days after administration [37], thus advocating that the cumulative dose and most likely the possibility of adaptation of the infant population may be determinant for de novo GSH synthesis. The levels of MDA, a marker of lipid peroxidation, were evaluated in the cumulative dose of 7.0 mg/kg in infant and adult mice, and no statistically significant changes were observed. Our data corroborate that, although MTX clearly induces cardiac damage, it has a low capacity to induce oxidative stress and lipid peroxidation. In fact, in our work, MTX (6.0 mg/kg) did not alter the expression of SOD2/MnSOD but significantly increased the levels of catalase in the heart of both adult and infant mice. Since catalase plays an important role in breaking down H_2_O_2_ and maintaining cellular redox homeostasis, this increase may be due to the adaptation of the cells to damage caused by MTX. In fact, in low mitochondrial oxygen consumption, an increase of H_2_O_2_ production occurred in skeletal muscle, which was accompanied by fatty acids β-oxidation. Nevertheless, catalase transfection and overexpression led to the normalization of energetic pathways [51]. Furthermore, and regarding glutathione peroxidase, its expression increased significantly in the heart of infant mice, 17 days after the last administration, suggesting that the heart is adjusting to MTX and that could give infant mice more protection concerning the MTX-induced oxidative injuries. That protection can be seen as infants had less histological damage than adults did. 

The general conviction is that in in vivo models, MTX does not increase lipid peroxidation, serum lipids, or significantly alter the redox status of the heart, indicating that oxidative stress does not play a major role in MTX-induced cardiac damage [37,41,52]. Even so, a recent work by our group showed that MTX might increase protein oxidative damage [53]. Herein, protein carbonylation increased significantly in the heart of adult mice (6.0 mg/kg) exposed to MTX, suggesting that MTX is able to cause permanent changes in proteins. Protein carbonylation may lead to protein partial or total function inactivation, with a wide range of downstream functional consequences, subsequent cellular dysfunction, and tissue damage [54]. A previous work also showed that adult mice treated with a 9.0 mg/kg cumulative dose of MTX had increased heart protein carbonylation, 24 h after the last administration, while in infants those changes were not seen in the heart [34]. Once again, infants seemed more protected towards MTX-inflicted damage. Whether that happens because they are able to activate antioxidant defences, as seen earlier, or they have higher intrinsic proteasome activity, needs to be further looked at.

Despite the low impact of MTX at 6.0 mg/kg dose on oxidative stress markers, cardiac immunohistochemical analysis revealed that MTX significantly increased the expression the NF-κB p65 subunit. Subsequently, a Western blot analysis indicated that the MTX-treated adult mice showed a tendency for increased expression of NF-κB p65 levels (*p* = 0.08), the canonical NF-κB pathway, and significantly enhanced the expression of NF-κB p52 levels, the noncanonical pathway of this transcription factor. Activation of the canonical NF-κB pathway provides rapid responses (within minutes) [55]. In contrast, the noncanonical pathway is comparatively slower (activated within hours) and can be sustained for longer periods [55]. NF-κB plays an important role in regulating inflammatory response and its activation alters the phenotype of macrophages [56]. In this study, the MTX-treated adult mice showed a higher density of infiltrating M1 macrophages. Macrophages have an important role in the innate immune system and have been described to be relevant cells within the cardiovascular system [57,58]. The increase of cardiac macrophages in adult mice can suggest that upon MTX-induced lesions, an immunologic response is induced, resulting in infiltration of inflammatory cells. M1 macrophages are involved in pro-inflammatory responses and following activation, they produce pro-inflammatory cytokines such as IL-1, IL-6, IL-12, and TNF-α, and chemokines [56]. Notably, MTX administration significantly altered the expression of pro-inflammatory cytokines, increasing TNF-α and decreasing IL-6 in mice cardiac tissue, reaching statistical significance in adult mice. The 26S proteasome activity has a role in substantially augmenting the generation of pro-inflammatory cytokines, although immunoproteasome activity differs with aging [59]. Whether differences in the activity of proteasome on different ages implicate differential results seen in protein carbonylation and in cytokine levels needs to be determined in more detail. Nonetheless, the pro-inflammatory cytokines such as TNF-α and IL-6, are responsible for the development and progression of cardiovascular diseases [60]. TNF-α has been implicated in patients with newly diagnosed HF, where increased levels are associated with ventricular hypertrophy, ventricular dilatation, interstitial infiltrates, interstitial fibrosis, rare myocyte apoptosis, a diminished ejection fraction, attenuation of β_1_-adrenergic responsiveness, and expression of atrial natriuretic factor in the left ventricle [21]. IL-6 is involved in inflammation but reveals additional cardiovascular properties through the regulation of cardiomyocyte hypertrophy and apoptosis [61]. Under stressful conditions, namely excessive hemodynamic stimulation and mechanical stress, cytokines promote and sustain an unbalanced status within the myocardium, with enhanced production of TNF-α accompanied by a simultaneous reduction of IL-6 levels and insulin-like growth factor (IGF)-1, that may critically contribute to the development of the HF phenotype [62]. Doxorubicin is an anthracycline often used in cancer that shares similar clinical cardiotoxicity with MTX. It has been shown that doxorubicin caused a significant increase in the production of pro-inflammatory interleukins (IL-8, IL-6, and IL-1β) in vitro, showing the possible importance of inflammation on doxorubicin-induced cardiotoxicity [63]. Moreover, nanoemulsions loaded with anti-inflammatory nutraceuticals inhibited the secretion of inflammatory cytokines IL-6, IL-8, IL-1β, and TNF-α from cardiac cells incubated with doxorubicin, suggesting that anti-inflammatory drugs activated molecular mechanisms of cardioprotection during doxorubicin treatment [64]. Nevertheless, the inflammatory pathways are also affected by immune checkpoint inhibitors, nivolumab and ipilimumab, that caused changes in NLRP3 inflammasome, MyD88 complex NF-κB/p65 expression, and in several interleukins in co-cultures of lymphocytes with tumor or with cardiac cells. Those changes in several inflammatory signals were also seen in the heart after ipilimumab in an in vivo model [65]. While in peripheral blood mononuclear cells of secondary progressive MS patients, MTX was reported to not seemly influence the production of inflammatory mediators, IL-6, IL-12p40, IL-10, and TGF-β [66], our data shows other results in the heart. Our data may in fact reveal other pathways of MTX-induced cardiotoxicity.

## 4. Materials and Methods

β-Nicotinamide adenine dinucleotide 2′-phosphate reduced (NADPH) tetrasodium salt hydrate was acquired from PanReac AppliChem ITW Reagents (Barcelona, Spain). Ethylenediaminetetraacetic acid (EDTA), perchloric acid (HClO_4_), sodium hydroxide (NaOH), magnesium chloride (MgCl_2_), sodium carbonate (Na_2_CO_3_), disodium phosphate (Na_2_HPO_4_), copper (II) sulphate (CuSO_4_), potassium bicarbonate (KHCO_3_), potassium dihydrogen phosphate (KH_2_PO_4_), magnesium sulphate (MgSO_4_), potassium chloride (KCl), Histosec paraffin pastilles, and FolinCiocalteu reagent, mouse monoclonal dinitrophenyl (DNP)-KLH (MAB2223) were purchased from Merck (Darmstadt, Germany). Bio-Rad DC protein assay kit was purchased from Bio-Rad Laboratories (Hercules, CA, USA). Phosphate buffered saline solution (PBS) was purchased from Biochrom (Berlin, Germany), sodium chloride (NaCl), and sodium dodecyl sulphate (SDS) from VWR (Leuven, Belgium), potassium sodium tartrate from Fluka (Buchs SG, Switzerland), methanol, DPX mounting media, and xylene from Thermo Fisher Scientific (Loughborough, UK). Harris haematoxylin was purchased from Harris Surgipath (Richmond, IL, USA), eosin 1% aqueous from Biostain (Traralgon, Australia), and Isoflurane (Isoflo^®^) was obtained from Abbott Animal Health (North Chicago, IL, USA). ABX Pentra reagents were purchased from HORIBA (Kyoto, Japan). Enhanced chemiluminescence (ECL) reagents, and 0.45 µm Amersham Protran nitrocellulose blotting membrane were purchased from GE Healthcare Bio-Sciences (Pittsburgh, PA, USA). Mouse monoclonal anti-ATP synthase subunit beta (ab14730), rabbit polyclonal anti- GAPDH(ab9485), rabbit polyclonal anti-IL-6 (ab83339), rabbit polyclonal anti-NF-κB p65 (ab16502), mouse monoclonal anti- NF-κB p100/p52 (ab71108), rabbit polyclonal anti-TNF-α (ab66579), rabbit polyclonal anti-SOD2/MnSOD (ab13534), rabbit polyclonal anti-catalase (ab16731), rabbit polyclonal anti-glutathione peroxidase 1 (ab22604), rabbit polyclonal anti-myeloperoxidase (ab139748), rabbit polyclonal anti-mannose receptor (M2 macrophage, ab64693), rabbit polyclonal anti-CD68 (M1 macrophage, ab125212), goat anti-rabbit IgG-horseradish peroxidase (ab97051) and rabbit anti-mouse IgG-horseradish peroxidase (ab6728) from Abcam (Cambridge, UK). 3,3’-Diaminobenzidine (DAB), HIGHDEF^®^ DAB chromogen/substrate set was purchased from Enzo Life Sciences (Miraflores, Portugal). Water was purified with a Milli-Q Plus ultrapure water purification system (Millipore, Bedford, Massachusetts, USA). All the other reagents used were purchased from Sigma Aldrich (St. Louis, MO, USA).

### 4.1. Animals

Male CD-1 mice (Mus Musculus) were acclimated for a week before experiments began and were accompanied by experienced veterinarians during all experimental procedures. Temperature (22 ± 2 °C) and humidity (50 ± 10%) were maintained at appropriate intervals, during 12-hour light–dark cycles. The animals were kept in groups of 3 per cage, with environmental enrichment always present. The mice were maintained on a rodent diet (with 4RF21 GLP certificate diet from Mucedola, Settimo Milanese, Italy) and with autoclaved water given ad libitum according to the guidelines defined by the European Council Directive (2010/63/EU) transposed into Portuguese legislation (Decreto-Lei no. 113/2013). Considering the animals’ welfare, a “scoring system” was created (Table S1) to evaluate and minimize their suffering and stress. All experiments with animals were handled with the approval of the local animal welfare body (ICBAS-UP ORBEA) and the Portuguese national authority for animal health (DGAV, processes no. 0421/000/000/2013 and 0421/000/000/2016). 

### 4.2. Experimental Protocol

Male CD-1 mice have been considered a good model to mimic the effects of MTX therapy in two different life stages [34]. Herein, infants and adult mice were used: the mice in the infant group weighed 10–12 g and were 3–4 weeks old, while mice in the adult group weighed 38–56 g and were 8–12 weeks old. According to the literature, infant mice had not entered puberty (equivalent to children), since in this stage of development 1 human year corresponds to 56.77 mice days [67,68], meaning that at the beginning of the administrations, the age of infant mice corresponded to roughly 135–180 human days. On the other hand, the mice at 8 to 12 weeks old clearly reached the young adulthood phase [67,68], since adult mice reach sexual maturity on average at 10 weeks, while in humans, this stage corresponds to an average age of 20 years [67,68].

### 4.3. Administration Schedule

Since MTX is given in cycles and throughout multiple administrations in humans, the experiments herein enclosed aimed to mimic human MTX-therapy, and drug administration was interrupted by free-drug periods [34]. Furthermore, allometric scaling was used to ensure that the cumulative dose of MTX administered did not exceed the maximum MTX recommended in humans [69,70], as to maintain clinical relevance. Two cumulative doses were given: 6.0 mg/kg MTX and 7.0 mg/kg MTX. In infants (to convert dose in mg/kg to dose in mg/m^2^, we multiply by Km factor 25), it corresponded in human equivalent to 33.5 mg/m^2^ and 39.1 mg/m^2^, respectively [69,70]. In adult mice (Km factor 37), the cumulative doses of 6.0 mg/kg MTX and 7.0 mg/kg MTX had a human equivalent of 36.3 mg/m^2^ and 42.3 mg/m^2^, respectively [69,70]. These calculations were estimated from the general body weight of infant (8.0 kg) and adult (70.0 kg) human population [69,70] at the ages calculated previously. 

The mice received IP administrations of MTX, in the afternoon period, to decrease MTX-induced lethality attributed to the daily circadian rhythm [35]. In humans, MTX is administered intravenously, but in mice, the IP route affords the same cardiac effects of MTX [34], while avoiding the high risk of extravasation of the endovenous route that can lead to tissue necrosis [71]. The doses were delivered through 6 IP injections (two per week), alternating between the left and right side of the abdomen in order to avoid extra injury and pain. MTX dihydrochloride was dissolved in sterile 0.9% saline solution. Control mice were injected with saline solution (0.9% NaCl) in the same volume and conditions as MTX-treated mice. During the experimental period, food and water intake, body weight, and animal welfare were assessed daily. 

### 4.4. Blood Collection and Plasma Biomarkers

Animals were anesthetized through the inhalation of 5% isoflurane and then sacrificed by exsanguination when fully sedated. After euthanasia, the animal’s abdominal cavity was exposed, and blood was collected in the inferior vena cava into EDTA-containing tubes. Blood was then centrifuged (920 g, 10 min, 4 °C), and the obtained plasma was stored at −20 °C for the determination of AST, ALT, CK–MB, and total-CK. Plasma biomarkers were determined through enzymatic assays in the apparatus ABX Pentra 400 with ABX Pentra reagents (Japan), according to the manufacturer’s instructions.

### 4.5. Tissue Collection

Hearts were removed, weighed, and processed as follows: (1) segments of heart that covered both the left and right ventricle were placed in 4% paraformaldehyde, pH 7.2–7.4, and used for histological and immunohistochemistry analysis; (2) cardiac sections of the same segments were placed in complete RIPA buffer (50 mM Tris-HCl, 150 mM NaCl, 1% Triton X-100 (*v*/*v*), 0.5% sodium deoxycholate (*w*/*v*), and 0.1% SDS (*w*/*v*), pH 8.0, supplemented with 0.25 mM phenylmethanesulfonyl fluoride (PMSF), 1 mM sodium metavanadate (Na_3_VO_4_), 10 mM sodium fluoride (NaF), 1 mM DL-dithiothreitol (DTT) and 0.5% (*v*/*v*) complete protease inhibitor cocktail and stored at −80 °C for immunoblotting analysis; and (3) the remaining heart was homogenized with an Ultra-Turrax^®^ homogenizer in ice-cold 0.1 M phosphate-buffered solution, pH 7.4. The homogenate was aliquoted for other determinations (namely protein determination by the Lowry method [72]) or placed to a final concentration of 5% HClO_4_ [for ATP, tGSH, GSH, and GSSG, catecholamines, and lipid peroxidation]. All steps were performed on ice.

### 4.6. Histological Analysis of Heart Tissue

All histological procedures were conducted according to previously published procedures [34]. Sections were subjected to haematoxylin and eosin for routine histological evaluation and Sirius red for fibrous tissue. The slides were examined and photographed with a Carl Zeiss Imager A1 light microscope equipped with an AxioCam MRc 5 digital camera (Oberkochen, Germany). Histopathological evidence of tissue damage was calculated according to its severity and incidence in every slide, as previously published [34]. Sections stained with Sirius red were used to assess collagen deposition, which was evaluated using ImageJ software (version 1.52a, http://imagej.nih.gov/ij, Wayne Rasband, NIH, Bethesda, MD, USA). The results of collagen content are expressed as a percentage of collagen fibres, as previously detailed [34].

### 4.7. Determination of Cardiac Noradrenaline Levels

In experiment 1, cardiac noradrenaline levels were determined, as described before [34]. For a more ascertain identification of the chromatographic peaks in heart samples, two independent procedures were used: comparison of retention time with pure standards and spiking the samples with standards of noradrenaline.

### 4.8. Measurement of ATP Levels

ATP levels were determined by a bioluminescent assay based on the luciferin-luciferase reaction, as previously described [34,73]. The results were expressed as nmol of ATP per mg of protein (nmol ATP/mg protein).

### 4.9. Determination of Cardiac Creatine and Phosphocreatine Levels

In experiment 1, cardiac creatine and phosphocreatine levels were evaluated, as described [34]. The phosphocreatine levels were calculated setting the difference between total creatine and free creatine.

### 4.10. Measurement of tGSH, GSH, and GSSG

tGSH and GSSG levels were evaluated by the DTNB-GSSG reductase recycling assay, as previously described [34,74]. The levels of GSH were calculated by the formula: GSH = tGSH−2 × GSSG. The results of tGSH, GSH, and GSSG were normalized to the total protein content and were expressed as nmol of GSH or GSSG per mg of protein (nmol GSH/mg protein or nmol GSSG/mg protein).

### 4.11. Assessment of Lipid Peroxidation

Lipid peroxidation was measured by assessing MDA values and comparing them with a standard curve of MDA after reaction with thiobarbituric acid (TBA). The analysis of MDA-equivalent content in standards and samples was performed by high-performance liquid chromatography, as previously described [34].

### 4.12. Protein Carbonylation by Slot Blot Analysis

Heart sections were lysed in RIPA buffer (supplemented with PMSF, DTT, NaF, Na_3_VO_4,_ and a cocktail of protease inhibitors) through sonication and were kept at −80 °C until analysis. Samples containing 20 µg of protein (as assessed by the Bio-Rad DC Protein assay) were then processed as previously described [75]. Immunoreactive bands were detected, and digital images were acquired using the ChemiDoc Imaging System version 2.3.0.07 (Bio-Rad, Hercules, CA, USA). The obtained images were analysed with Image Lab software version 6.0.1 (Bio-Rad, Hercules, CA, USA).

### 4.13. Western Blotting Analysis

Heart tissue was lysed in a lysis buffer (RIPA supplemented with PMSF, DTT, NaF, Na_3_VO_4,_ and a cocktail of protease inhibitors). The amount of total protein was quantified using the Bio-Rad DC Protein assay, and then the total protein (20 µg from each sample) was fractionated by electrophoresis on a 12.5% SDS-PAGE. Gels were blotted onto a nitrocellulose membrane (Amersham Protran, GE Healthcare, Germany) in a transfer buffer (25 mM Tris, 192 mM glycine, pH 8.3, and 20% methanol) for 2 h (200 mA). Then, nonspecific binding was blocked with 5% (*w*/*v*) dry non-fat milk in TBS-T (100 mM Tris, 1.5 mM NaCl, pH 8.0 and 0.5% Tween 20). Membranes were incubated with primary antibody diluted 1:1000 (mouse anti-ATP synthase subunit beta, rabbit anti-GAPDH, mouse anti-NF-κB p100/p52, rabbit anti-NF-κB p65, rabbit anti-SOD2/MnSOD, rabbit anti-glutathione peroxidase 1) or 1:500 (rabbit anti-IL-6, rabbit anti-TNF-α, rabbit anti-catalase, and rabbit anti-myeloperoxidase) in 5% w/v non-fat dry milk in TBS-T, for 2 h at room temperature or overnight, washed and incubated with secondary horseradish peroxidase-conjugated anti-rabbit (1:10,000) or anti-mouse (1:5000). Immunoreactive bands were detected by enhanced chemiluminescence ECL (Amersham Pharmacia Biotech) according to the manufacturer’s indications. The immunoreactive bands were detected using the ChemiDoc Imaging System version 2.3.0.07 (Bio-Rad, Hercules, CA, USA). The obtained images were analysed with Image Lab software version 6.0.1 (Bio-Rad, Hercules, CA, USA). Protein loading was confirmed by the Ponceau S staining.

### 4.14. Immunohistochemistry

The detection of NF-κB subunit p65 and M1 and M2 macrophage markers was performed by immunohistochemistry in a protocol already described [76], where we made minor modifications. After heart tissue was deparaffinized, antigens were unmasked by the pressure cooker antigen-retrieval procedure: slides were immersed in 10 mM citrate buffer, pH 6.0, at 100 °C and were placed in a pressure cooker for 10 min and then cooled for 20–30 min. Following the blocking step, each slide was incubated with anti-NF-κB p65 polyclonal rabbit antibody (1:50), or anti-mannose receptor polyclonal rabbit antibody (M2 macrophage) (1:50), or anti-CD68 polyclonal rabbit antibody (M1 macrophage) (1:50) in PBS containing 0.05% Tween 20 (*v*/*v*) (PBS-T) overnight (4 °C). After washing four times (5 min each) with PBS, the sections were incubated for 2 h, at 37 °C with a goat anti-rabbit IgG-horseradish peroxidase secondary antibody (1:100) in PBS-T. The sections were then washed four times (5 min) under gentle stirring and incubated with DAB reagent for 1 min. After washing, the slides were counterstained with a solution of haematoxylin-water (1:11) for 3 min and once again washed. Finally, slides were mounted in DPX medium with coverslips and routine procedures followed. Negative controls were performed as described, with the omission of the primary antibody incubation step. All preparations were analysed in a Carl Zeiss Imager A1 light microscope and images were recorded with a coupled AxioCam MRc5 digital camera (Oberkochen, Germany). The semi-quantitative analysis of IHC images was made using ImageJ software (version 1.52a, http://imagej.nih.gov/ij, Wayne Rasband, NIH, Bethesda, MD, USA).

### 4.15. Statistical Analysis

Results are expressed as mean ± standard deviation (SD). Statistical analyses of animal weight, food, and water intake data were carried out by the two-way analysis of variance (two-way ANOVA) followed by the Sidak post hoc test. When two groups and one time point were analysed, the outliers were identified using the ROUT method (Q = 1%) first and then statistical analysis was performed by the Student’s t-test when the distribution was normal or by the Mann–Whitney test when the distribution was not normal. Statistical significance was considered when *p* values <0.05. To perform the statistical analysis, the GraphPad Prism 8.0 software program (San Diego, CA, USA) was used.

## 5. Conclusions

Inflammation plays a key role in the development and progression of cardiovascular diseases. To the best of our knowledge, this is the first time that inflammatory signals were shown to play an important role on MTX-induced cardiotoxicity. Overall, our work demonstrates inflammation in cardiac tissue even when no major changes were observed in the redox status and the ATP cardiac levels in mice exposed to lower doses of MTX. Moreover, MTX administration caused dissimilar responses in adult and infant mice, which may be linked to the highest antioxidant adaptation of infant or repair ability. Nonetheless, more research is warranted to investigate the involvement of inflammation on MTX-induced cardiotoxicity or if targeting specific mechanisms of the immune system will be advantageous to reverse cardiotoxicity of this anticancer drug.

## Figures and Tables

**Table 1:** Semi-quantitative analysis of morphological parameters (cellular degeneration, necrosis, inflammatory activity, and tissue disorganization) in the heart of 7.0 mg/kg and 6.0 mg/kg MTX-treated and controls groups, in adult and infant animals.

**Hematoxylin-eosin staining**				
	**INFANT**		**ADULT**	

	**Control**	**MTX 7.0 mg/kg**	**Control**	**MTX 7.0 mg/kg**
**Cellular Degeneration**	0.50 ± 0.54	0.82 ± 0.63 **	0.64 ± 0.63	1.42 ± 0.71 ****
**Necrosis**	0.00 ± 0.00	0.04 ± 0.20	0.00 ± 0.00	0.23 ± 0.43 ***
**Inflammatory Activity**	0.22 ± 0.42	0.85 ± 0.52 ****	0.30 ± 0.46	1.02 ± 0.49 ****

	**Control**	**MTX 6.0 mg/kg**	**Control**	**MTX 6.0 mg/kg**
**Cellular Degeneration**	0.07 ± 0.25	0.47 ± 0.51 ***	0.02 ± 0.13	1.00 ± 0.00 ****
**Necrosis**	0.00 ± 0.00	0.27 ± 0.45 **	0.00 ± 0.00	1.02 ± 0.14 ****
**Inflammatory Activity**	0.08 ± 0.27	0.50 ± 0.51 ***	0.05 ± 0.22	1.65 ± 0.49 ****

Results of haematoxylin–eosin staining, given in scores, are presented as mean ± SD and were obtained from 3 animals from each treatment group, with exception of the control in 7.0 mg/kg MTX-treated infant group of haematoxylin–eosin staining (*n* = 2). Statistical comparisons were made using the Mann-Whitney test: ** *p* < 0.01, *** *p* < 0.001, **** *p* < 0.0001, MTX vs. control.

**Table 2:** Semi-quantitative analysis of fibrosis in the heart of the 7.0 mg/kg and 6.0 mg/kg MTX-treated and respective controls groups, in adult and infant animals.

**Sirius Red Staining**				
	**INFANT**		**ADULT**	

	**Control**	**MTX 7.0 mg/kg**	**Control**	**MTX 7.0 mg/kg**
**% area ratio of collagen/skeletal muscle**	0.14 ± 0.07	0.17 ± 0.05	0.14 ± 0.02	0.24 ± 0.06 ****

	**Control**	**MTX 6.0 mg/kg**	**Control**	**MTX 6.0 mg/kg**
**% area ratio of collagen/skeletal muscle**	0.10 ± 0.03	0.15 ± 0.03	0.10 ± 0.02	0.16 ± 0.05 **

Results of Sirius red staining, given in area of collagen/skeletal muscle, are presented as mean ± SD and were obtained from 3 animals from each treatment group. Statistical comparisons were made using the Mann–Whitney test: ** *p* < 0.01, **** *p* < 0.0001, MTX vs. control.

**Table 3:** Biochemical cardiac parameters of the 7.0 mg/kg MTX-treated and control mice.

	INFANT		ADULT	
	**Control**	**MTX 7.0 mg/kg**	**Control**	**MTX 7.0 mg/kg**
**tGSH (nmol/mg protein)**	7.72 ± 1.18	9.41 ± 1.63 *	8.01 ± 0.77	8.57 ± 2.33
**GSSG (nmol/mg protein)**	0.62 ± 0.19	0.77 ± 0.15	0.60 ± 0.10	0.60 ± 0.34
**GSH/GSSG ratio**	11.94 ± 5.23	10.62 ± 2.01	11.84 ± 3.52	15.40 ± 8.63
**MDA (nmol/g protein)**	27.23 ± 9.23	28.42 ± 7.32	30.65 ± 7.59	34.83 ± 12.44
**ATP (nmol/mg protein)**	3.94 ± 1.97	3.77± 1.42	3.92 ± 1.34	3.23 ± 1.39
**Creatine (nmol/mg protein)**	74.77 ± 16.25	78.94 ± 11.09	82.17 ± 13.24	84.13 ± 25.30
**Phosphocreatine (nmol/mg protein)**	41.06 ± 9.71	43.08 ± 6.68	37.25 ± 8.11	49.10 ± 14.36 (*p* = 0.06)
**Noradrenaline (nmol/g protein)**	8.26 ± 5.18	9.03 ± 4.63	9.79 ± 1.99	4.64 ± 5.16 *

Results, in nmol/mg protein or nmol/g protein, are presented as mean ± SD and were obtained from 7 to 8 animals from each treatment group. Statistical analyses were made using the t-test for all adult groups, with the exception of total glutathione (tGSH) and reduced glutathione/oxidized glutathione ratio (GSH/GSSG), where the Mann–Whitney test was done. For the infant data, the Mann–Whitney test was used. * *p* < 0.05, MTX 7.0 mg/kg vs. control.

**Table 4:** Number of cells staining positive for activated macrophages marked as M1 and macrophage marked as M2 in the heart of MTX-treated and control groups, in adult and infant animals.

	INFANT		ADULT	
**Immunohistochemistry**				
	**Control**	**MTX 6.0 mg/kg**	**Control**	**MTX 6.0 mg/kg**
**M1 macrophage**	4.28 ± 3.03	9.33 ± 7.70	12.83 ± 15.37	68.11 ± 22.77 ****
**M2 macrophage**	66.61 ± 43.40	62.56 ± 24.92	113.70 ± 23.10	96.56 ± 28.33

Results were expressed as mean ± SD. The value of macrophages was obtained from three animals from each treatment group. Statistical comparisons were made using the t-test for M2 macrophage evaluation in the adult group and the Mann–Whitney test for all infant group and M1 macrophage of adult group: **** *p* < 0.0001, MTX 6.0 mg/kg vs. control. Three animals per treatment, and for each animal, six photos of different areas of cardiac tissue were evaluated and quantified using ImageJ software version 1.52a.

**Table 5:** Number of cells staining positive for activated NF-κB in the heart of MTX-treated and control groups, in adult and infant animals.

	INFANT		ADULT	
**Immunohistochemistry**				
	**Control**	**MTX 6.0 mg/kg**	**Control**	**MTX 6.0 mg/kg**
**NF-ĸB p65 cells**	28.00 ± 12.35	26.00 ± 8.62	56.28 ± 15.69	75.17 ± 17.05 **

Results were expressed in number of cells positive to NF-κB p65 and as mean ± SD. Results were obtained from three animals from each treatment group. Statistical comparisons were made using the t-test for adult group and the Mann–Whitney test for infant group: ** *p* < 0.01, MTX 6.0 mg/kg vs. control. Three animals per treatment, and for each animal, six photos of different areas of cardiac tissue were evaluated and quantified using ImageJ software version 1.52a.

## Data Availability

The data presented in this study are available on request from the corresponding author.

## Supplementary Materials

The following are available online at https://www.mdpi.com/article/10.3390/ph14060510/s1, Figure S1. [A, B] Body weight in [A] infant and [B] adult mice exposed to a cumulative dose of 7.0 mg/kg MTX. [C and D] Water consumption of [C] infant and [D] adult mice exposed to a cumulative dose of 7.0 mg/kg MTX. [E, F] Food consumption of [E] infant and [F] adult mice exposed to a cumulative dose of 7.0 mg/kg MTX. Results are presented in grams (g) of food intake/day/weight of animal, mL of water intake/day/weight of animal, or g of body weight, and as mean ± standard deviation (SD), from 8 per group. Statistical comparisons were made using two-way ANOVA followed by the Sidak’s post hoc test (* *p* < 0.05, ** *p* < 0.01, *** *p* < 0.001, and **** *p* < 0.0001, MTX 7.0 mg/kg vs. control). Figure S2. [A and B] Body weight in [A] infant and [B] adult mice exposed to a cumulative dose of 6.0 mg/kg MTX. [C and D] Water consumption of [C] infant and [D] adult mice exposed to a cumulative dose of 6.0 mg/kg MTX. [E and F] Food consumption of [E] infant and [F] adult mice exposed to a cumulative dose of 6.0 mg/kg MTX. Results are presented in grams (g) of food intake/day/weight of animal, mL of water intake/day/weight of animal, or g of body weight, and as mean ± SD, from 6 per infant group and 17 per adult group. Statistical comparisons were made using two-way ANOVA followed by the Sidak’s post hoc test * *p* < 0.05, ** *p* < 0.01, *** *p* < 0.001, and **** *p* < 0.0001, MTX 6.0 mg/kg vs. control). Figure S3. Loading control of Ponceau S staining of [A] ATP synthase subunit beta (52 kDa) and glyceraldehyde-3-phosphate dehydrogenase (GAPDH) (37 kDa), [B] Superoxide dismutase 2/manganese-dependent superoxide dismutase (SOD2/MnSOD) (26.6 kDa) and Nuclear kappa B nuclear transcription factor (NF-κB) p65 (60 kDa), [C] catalase (60 kDa) and [D] glutathione peroxidase (22 kDa), [E] Tumour necrosis factor-α (TNF- α) (25 kDa), [F] interleukin-6 (IL-6) (24 kDa), [G] myeloperoxidase (48 kDa), [H] NF-κB p52 (50 kDa) from infant mice exposed to a cumulative dose of 6.0 mg/kg MTX or control animals. Figure S4. Loading control of Ponceau S staining of [A] ATP synthase subunit beta (52 kDa), glyceraldehyde-3-phosphate dehydrogenase (GAPDH) (37 kDa) and interleukin-6 (IL-6) (24 kDa), [B] catalase (60 kDa) and glutathione peroxidase (22 kDa), [C] Tumour necrosis factor-α (TNF-α) (25 kDa), [D] myeloperoxidase (48 kDa), [E] Superoxide dismutase 2/manganese-dependent superoxide dismutase (SOD2/MnSOD) (26.6 kDa) and Nuclear kappa B nuclear transcription factor (NF-κB) p52 (50 kDa) and [F] NF-κB p65 (60 kDa) from adult mice exposed to a cumulative dose of 6.0 mg/kg MTX or control animals. Table S1. General distress scoring sheet implemented for the evaluation of the general welfare of mice during all the experiment. Table S2. Plasma biomarkers and heart weight/body weight ratio after MTX (7.0 mg/kg cumulative dose). Table S3. Plasma biomarkers and heart weight/body weight ratio after MTX (6.0 mg/kg cumulative dose).
