# Supplementary material for: Inflammation as a Possible Trigger for Mitoxantrone-Induced Cardiotoxicity: An In Vivo Study in Adult and Infant Mice
Source: Pharmaceuticals (Basel). 2021 May 26;14(6):510. doi: 10.3390/ph14060510 (PMC8229902; doi:10.3390/ph14060510)
Supplement: Supplementary file 1 [file pharmaceuticals-14-00510-s001.zip › pharmaceuticals-1200205-supplementary.pdf]

Supplementary Materials

Article

Inflammation as a Possible Trigger for Mitoxantrone-induced Cardiotoxicity: an in vivo study in adult and infant mice

Ana Reis-Mendes [1,*], José Luís Dores-Sousa [1], Ana Isabel Padrão [2], Margarida Duarte-Araújo [3,4], José Alberto Duar-te [2,5], Vítor Seabra [5], Salomé Gonçalves-Monteiro [6,7], Fernando Remião [1], Félix Carvalho [1], Emília Sousa [8,9], Maria Lour-des Bastos [1], and Vera Marisa Costa [1,*]

[1] UCIBIO, REQUIMTE, Laboratory of Toxicology, Department of Biological Sciences, Faculty of Pharmacy, University of Porto, 4050-313 Porto, Portugal; joseldsousa@gmail.com (J.L.D.-S.); remiao@ff.up.pt (F.R.); felixdc@ff.up.pt (F.C.); mlbastos@ff.up.pt (M.L.B.)
[2] Research Center in Physical Activity, Health and Leisure (CIAFEL), Laboratory for Integrative and Translational Research in Population Health (ITR), Faculty of Sport, University of Porto, 4200-450 Porto, Portugal; apadrao@fade.up.pt (A.I.P.); jarduarte@fade.up.pt (J.A.D.)
[3] LAQV/REQUIMTE, University of Porto; 4050-313 Porto, Portugal; mdcma@icbas.up.pt (M.D.-A.)
[4] Department of Immune-Physiology and Pharmacology, Institute of Biomedical Sciences Abel Salazar, Uni-versity of Porto, 4050-313 Porto, Portugal
[5] IINFACTS-Institute of Research and Advanced Training in Health Sciences and Technologies, University Institute of Health Sciences (IUCS), CESPU, 4585-116 Paredes, Portugal; vitor.seabra@iucs.cespu.pt (V.S)
[6] LAQV/REQUIMTE, Laboratory of Pharmacology, Department of Drug Sciences, Faculty of Pharmacy, University of Porto, 4050-313 Porto, Portugal; salomemonteiro8180@gmail.com (S.G.-M.)
[7] MOREHealth, Outcomes Research Lab, Portuguese Institute of Oncology at Porto Francisco Gentil (IPO Porto), 4200-072 Porto, Portugal
[8] Laboratory of Organic and Pharmaceutical Chemistry, Chemistry Department, Faculty of Pharmacy, University of Porto, 4050-313 Porto, Portugal; esousa@ff.up.pt (E.S.)
[9] CIIMAR—Interdisciplinary Centre of Marine and Environmental Research, 4450-208 Porto, Portugal
* Correspondence: afreis.mendes@gmail.com (A.R.-M.); Tel.: +351 220 428 599; veramcosta@ff.up.pt (V.M.C.); Tel.: +351 220 428 599

Table S1. Scoring sheet for evaluation of the general welfare of mice during the experimental protocol.

	Clinical symptoms assessed	Score
**Attitude/ Activity**	Alert and active animal, normal posture and coat	0
	Some apathy, immobility, decrease grooming	1
	Apathy, chromodacriorea, loss of grooming	2
	Immobility, isolation, abnormal posture, chromodacriorea, piloerection	3
	Animal in decubitus, little reaction	4
**Weight loss/ stool consistency/ Bleeding**	Normal growth (infant)/ normal weight (adult), normal stool consistency and no bleeding	0
	Does not grow according to the curve (infant)/ maintains weight (adult), soft stool consistency and no bleeding	1
	Growth below the curve (infant)/ weight decrease ~ 5%/ 48h (adult); very soft	2

	stool; slight bleeding	
	Zero growth (infant)/ weight decrease ~ 10%/ 24h (adult), dehydration ~ 5%; fluid stools; slight bleeding	3
	Weight decrease (infant)/ weight decrease > 15%/ 24h (adult), dehydration ~ 8%; watery diarrhea; gross bleeding	4
**Body condition and ascites**	Normal condition and absence of ascites	0
	Presence of sizable abdominal enlargement or ascites; possessing a volume of ascites (g)/body weight (g) of 1-5%	1
	Presence of sizable abdominal enlargement or ascites; possessing a volume of ascites (g)/body weight (g) of > 5%	2
	Presence of sizable abdominal enlargement or ascites; possessing a volume of ascites (g)/body weight (g) of > 10 %	3
	Sizable abdominal enlargement or ascites with loss of a righting reflex; possessing a volume of ascites (g)/body weight (g) of > 15 %	4

Specific score: 3-4 in one specific parameter = urgent evaluation;

Total Score: 6-9 = daily evaluation in multiple occasions; 9-12 = sacrifice and sample collection.

	**Grimace Scale**	**Score**
**Orbital tightening**	Closing of the eyelid (narrowing of the orbital area)	0
	A wrinkle may be visible around the eye	1
	As a guideline, any eye closure that reduces the eye size by more than half should be coded as a "2".	2
**Nose bulge**	Bulging on the bridge of the nose	0
	Vertical wrinkles on the side of the nose	1
	In frontal headshots, a bulge may be seen as a widening of the nose area.	2
**Check bulge**	Bulging on the cheeks	0
		1
		2
**Ear position**	Ears rotate outwards and/or back, away from the face	0
	Ears may fold to form a "pinhead" shape	1
	Space between the ears increases	2
**Whisker change**	Whiskers are either pulled against the cheek, or pulled forward to "stand on end"	0
	Whiskers may clump together	1
	Whiskers lose their natural "downward" curve	2

Legend: 0: Not present, normal; 1: Moderate; 2: Obvious discomfort/pain

	**Intraperitoneal administration**	**Score**
**Response**	No vocalization or general discomfort (GD)/ no abdominal pain (AP)	0
	Does not vocalize, GD (transient immobility, local irritation); AP < 5 minutes	1
	Vocalize, evident GD; AP < 10 minutes complete/ reversed with analgesia	2
	Vocalize, evident GD; AP > 10 minutes complete/ reversed with analgesia	3

Figure S1

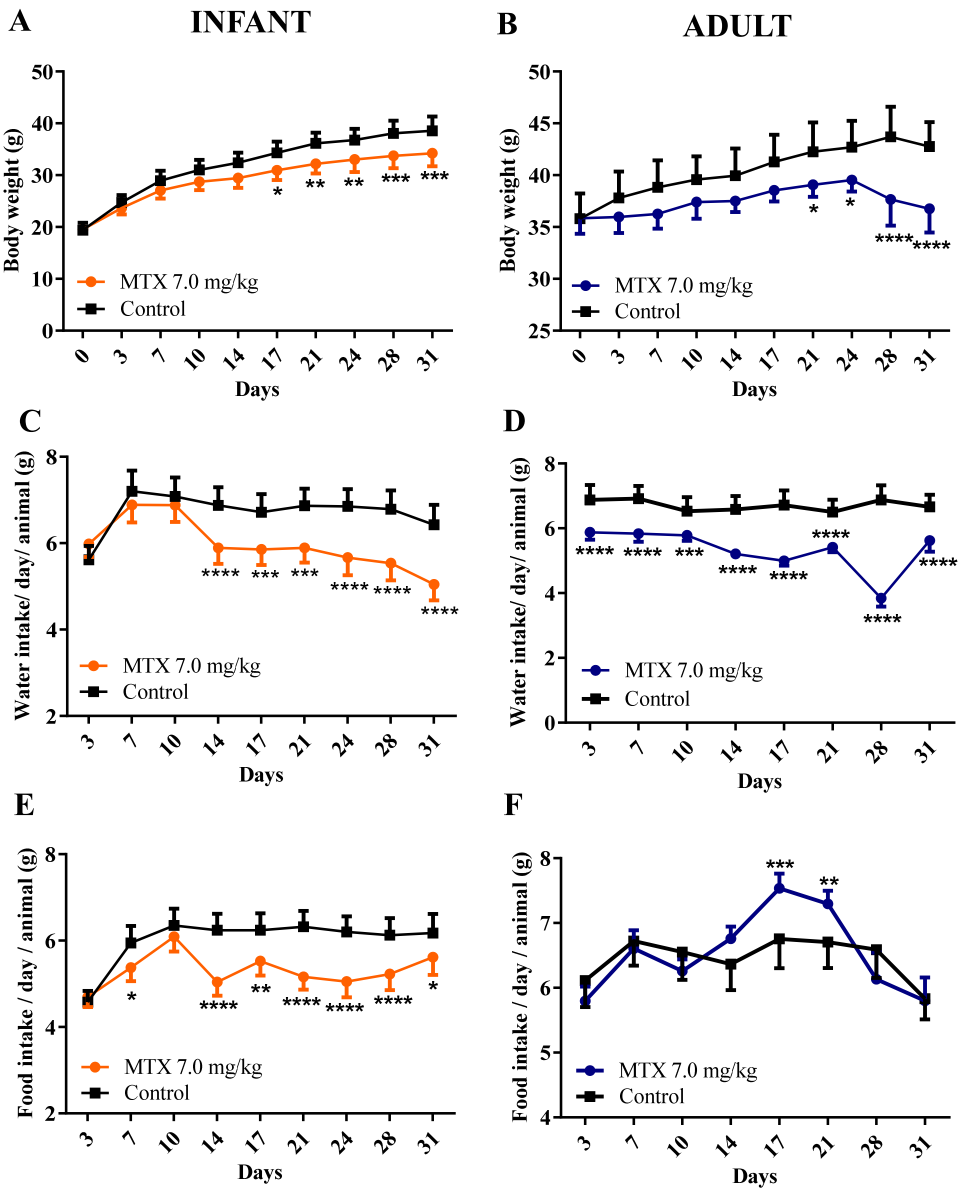

Figure S1. [A and B] Body weight in [A] infant and [B] adult mice exposed to a cumulative dose of 7.0 mg/kg MTX. [C and D] Water consumption of [C] infant and [D] adult mice exposed to a cumulative dose of 7.0 mg/kg MTX. [E and F] Food consumption of [E] infant and [F] adult mice exposed to a cumulative dose of 7.0 mg/kg MTX. Results are presented in grams (g) of food intake/day/weight of animal, mL of water intake/day/weight of animal or g of body weight, and as means ± standard deviation (SD), from 8 per group. Statistical comparisons were made using two-way ANOVA followed by the Sidak´s *post hoc* test (*p <0.05, **p < 0.01, ***p < 0.001, and ****p < 0.0001, MTX 7.0 mg/kg vs. control).

Figure S2

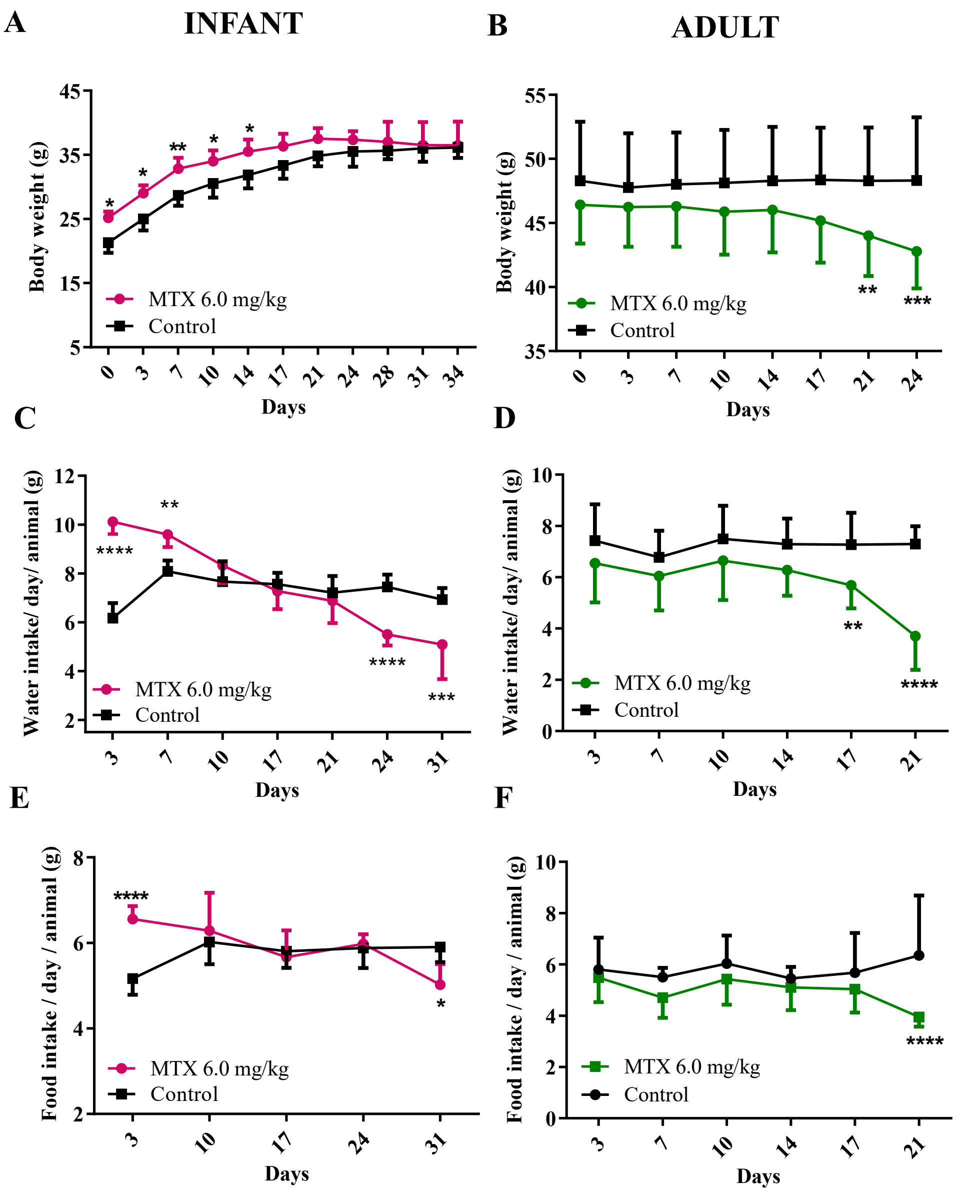

Figure S2. [A and B] Body weight in [A] infant and [B] adult mice exposed to a cumulative dose of 6.0 mg/kg MTX. [C and D] Water consumption of [C] infant and [D] adult mice exposed to a cumulative dose of 6.0 mg/kg MTX. [E and F] Food consumption of [E] infant and [F] adult mice exposed to a cumulative dose of 6.0 mg/kg MTX. Results are presented in grams (g) of food intake/day/weight of animal, mL of water intake/day/weight of animal or g of body weight, and as means ± SD, from 6 per infant group and 17 per adult group. Statistical comparisons were made using two-way ANOVA followed by the Sidak´s *post hoc* test (*$p < 0.05$, **$p < 0.01$, ***$p < 0.001$, and ****$p < 0.0001$, MTX 6.0 mg/kg vs. control).

Table S2. Plasma biomarkers and heart weight/body weight ratio after MTX (7.0 mg/kg cumulative dose).

	INFANT		ADULT	
**Plasma**				
	**Control**	**MTX 7.0**	**Control**	**MTX 7.0 mg/kg**
**AST(U/L)**	51.33 ± 8.31	70.20 ± 17.92	65.75 ± 21.79	87.13 ± 32.46
**ALT (U/L)**	37.00 ± 15.66	26.75 ± 3.30	39.63 ± 22.75	46.00 ± 37.04
**AST/ALT ratio**	1.69 ± 0.54	2.49 ± 0.40	1.80 ± 0.42	2.46 ± 0.91 (p = 0.06)
**CK-MB (U/L)**	73.67 ± 0.6.56	91.80 ± 14.96*	73.50 ± 33.12	99.25 ± 24.33
**Total-CK (U/L)**	45.14 ± 7.67	54.75 ± 4.03	78.38 ± 25.01	192.00 ± 141.93*
**Heart**				
**Heart weight/ body weight ratio (%)**	0.46 ± 0.04	0.45 ± 0.07	0.47 ± 0.05	0.42 ± 0.03*

Data of creatine-kinase - MB (CK-MB), total creatine-kinase (total CK), AST and ALT levels, in units per liter (U/L), are presented as means ± SD and were obtained from 4-8 animals of each group. Data of heart weight/body weight ratio, in percentage, are presented as means ± SD, and were obtained from seven (infant) and eight (adult) animals from each treatment group. Statistical comparisons were made using the t-test for CK-MB, total-CK and heart weight/body weight ratio in the adult group and the Mann-Whitney test for all other groups. *$p < 0.05$, MTX 7.0 mg/kg vs. control.

Table S3. Plasma biomarkers and heart weight/body weight ratio after MTX (6.0 mg/kg cumulative dose).

	INFANT		ADULT	
**Plasma**				
	**Control**	**MTX 6.0 mg/kg**	**Control**	**MTX 6.0 mg/kg**
**AST(U/L)**	37.67 ± 6.02	36.33 ± 20.62	58.00 ± 17.25	56.38 ± 20.63
**ALT (U/L)**	9.33 ± 4.27	34.52 ± 9.83**	25.60 ± 9.96	26.88 ± 8.52
**AST/ALT ratio**	4.81 ± 2.37	1.23 ± 0.23**	2.36 ± 0.66	2.27 ± 0.96
**CK-MB (U/L)**	-	-	77.22 ± 19.64	77.22 ± 16.82
**Total-CK (U/L)**	-	-	45.40 ± 29.77	58.00 ± 32.18
**Heart**				
**Heart weight/ body weight ratio (%)**	0.52 ± 0.04	0.47 ± 0.03	0.65 ± 0.13	0.63 ± 0.15

Data of CK-MB, total CK, AST and ALT levels, in units per liter (U/L), are presented as means ± SD and were obtained from six (infant) and 17 (adult) animals from each group. Data of heart weight/body weight ratio, in percentage, are presented as means ± SD, and were obtained from six (infant) and 17 (adult) animals from each treatment group. Statistical comparisons were

made using the t-test for CK-MB, ALT and AST/ALT ratio of adult group and the Mann-Whitney test for all other experiments $^{**}p < 0.01$, MTX 6.0 mg/kg vs. control.

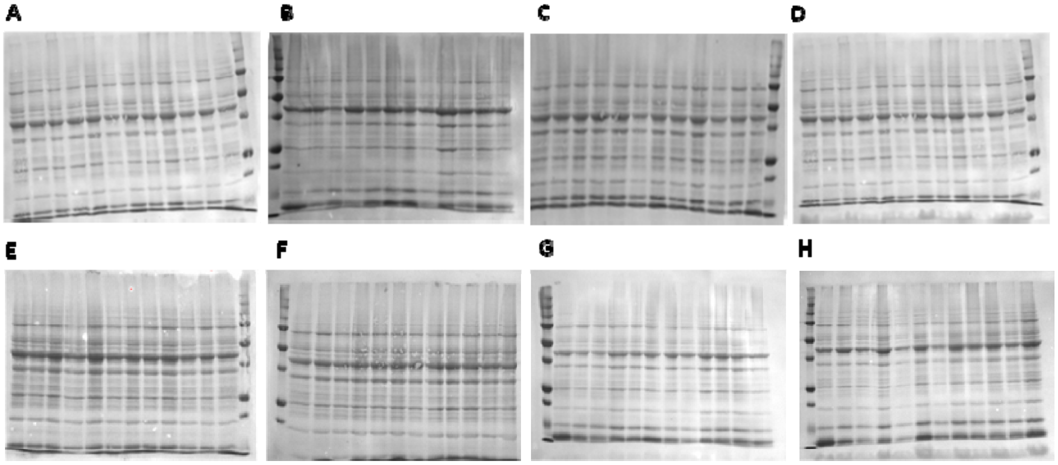

Figure S3. Loading control of Ponceau S staining of [A] ATP synthase subunit beta (52 kDa) and glyceraldehyde-3-phosphate dehydrogenase (GAPDH) (37 kDa), [B] Superoxide dismutase 2/ manganese-dependent superoxide dismutase (SOD2/MnSOD) (26.6 kDa) and Nuclear kappa B nuclear transcription factor (NF-κB) p65 (60 kDa), [C] catalase (60 kDa) and [D] glutathione peroxidase (22 kDa), [E] Tumour necrosis factor-α (TNF-α) (25 kDa), [F] interleukin-6 (IL-6) (24 kDa), [G] myeloperoxidase (48 kDa), [H] NF-κB p52 (50 kDa) from infant mice exposed to a cumulative dose of 6.0 mg/kg MTX or control animals.

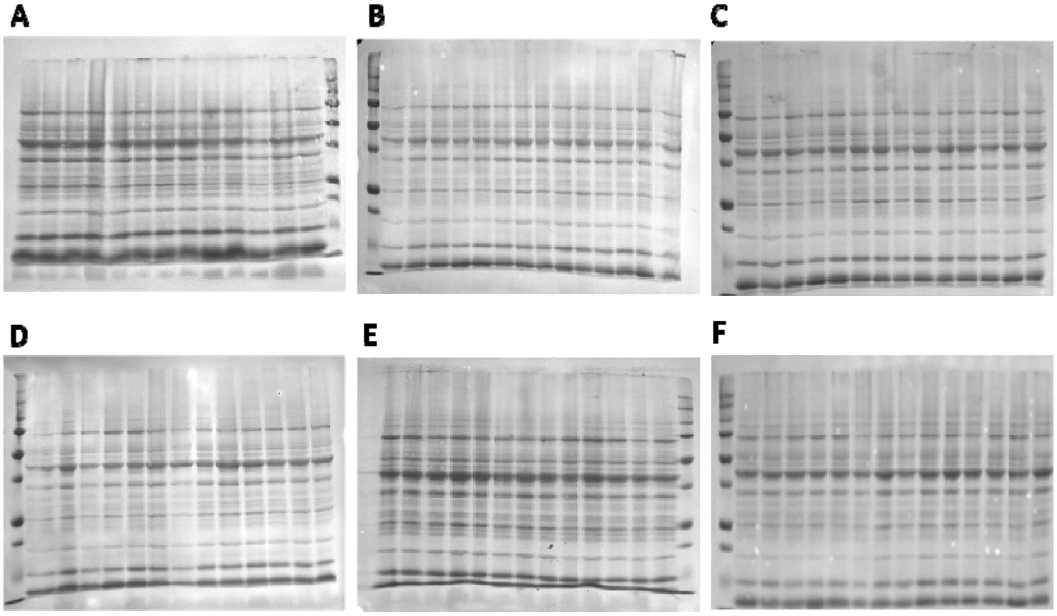

Figure S4. Loading control of Ponceau S staining of [A] ATP synthase subunit beta (52 kDa), glyceraldehyde-3-phosphate dehydrogenase (GAPDH) (37 kDa) and interleukin-6 (IL-6) (24 kDa), [B] catalase (60 kDa) and glutathione peroxidase (22 kDa), [C] Tumour necrosis factor-α (TNF-α) (25 kDa), [D] myeloperoxidase (48 kDa), [E] Superoxide dismutase 2/ manganese-dependent superoxide dismutase (SOD2/MnSOD) (26.6 kDa) and Nuclear kappa B nuclear transcription factor (NF-κB) p52 (50 kDa) and [F] NF-κB p65 (60 kDa) from adult mice exposed to a cumulative dose of 6.0 mg/kg MTX or control animals.
